# Rh(iii)-catalyzed building up of used heterocyclic cations: facile access to white-light-emitting materials†

**DOI:** 10.1039/d4sc02188f

**Published:** 2024-06-11

**Authors:** Jingxian Zhang, Tao Sun, Kangmin Wang, Ruike Hu, Chunlin Zhou, Haibo Ge, Bijin Li

**Affiliations:** a Chongqing Key Laboratory of Natural Product Synthesis and Drug Research, School of Pharmaceutical Sciences, Chongqing University Chongqing 401331 P. R. China bijinli@cqu.edu.cn; b Department of Chemistry and Biochemistry, Texas Tech University Lubbock TX 79409-1061 USA Haibo.Ge@ttu.edu

## Abstract

The first example of rhodium-catalyzed nondirected C–H activation/annulation reactions for the construction of fused heterocyclic cations is reported herein with excellent regioselectivity. Deuterium-labeling experiments indicated that the C(sp^3^)–H bond cleavage of the *N*-methyl group might be the rate-limiting step during the reaction process. This protocol provides an opportunity to rapidly access highly π-conjugated fused heterocyclic cations, which opens up a new avenue for efficient screening of single-molecular white-light-emitting materials, pure red-light-emitting materials, and π-conjugated radical materials. Importantly, novel white-light-emitting materials exhibited distinct anti-Kasha dual-emission and could rapidly be fabricated into robust organic and low-cost white light-emitting diodes.

## Abstract 2

This work unlocks an opportunity to rapidly construct fused heterocyclic cations for efficient screening of single-molecular white-light-emitting materials and pure red-light-emitting materials.

## Graphical abstract

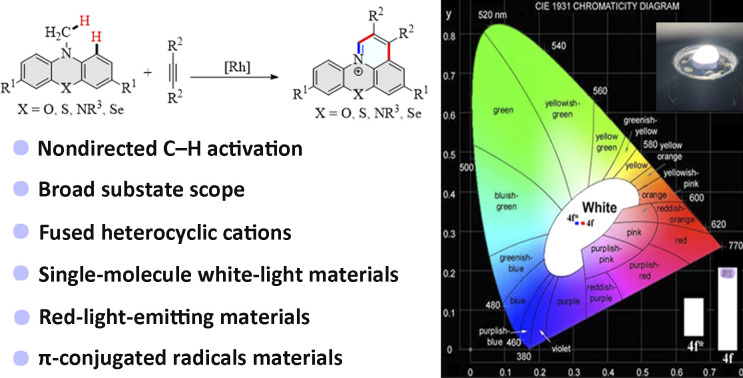

## Introduction

Fused heterocyclic cations have attracted considerable attention in scientific and engineering arenas due to their charming thermal, mechanical, optical, magnetic, electronic, and electrochemical properties.^1–17^ As such, fused heterocyclic cation materials have been widely applied in fluorescent bioimaging reagents, DNA intercalators, photosensitizers, optical devices, and electronic devices.^1–17^ Traditional synthetic methods for fused heterocyclic cations often suffer from tedious multiple-step syntheses or the use of toxic or environmentally harmful chemicals, and are thus environmentally and economically unbeneficial.^7,18–21^ Doubtlessly, the development of more atom- and step-economic syntheses of fused heterocyclic cation materials is highly desirable.

**Fig. 1:**
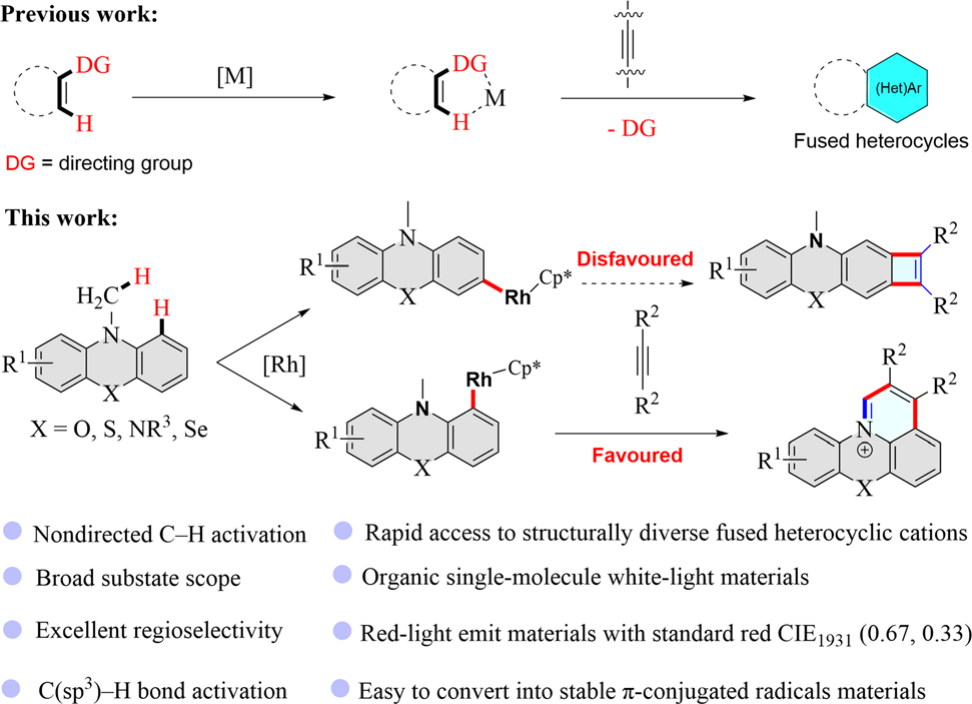
Metal-catalyzed C–H activation/annulation with alkynes to construct π-conjugated heterocycles. DG = directing group.

In the past decade, transition metal-catalyzed C–H functionalized has emerged as a powerful synthetic method and has aroused great interest in chemistry research because this strategy usually uses lower toxic reagents and can offer more atom- and step-economic syntheses of organic functionalization molecules than traditional synthetic methods, and could even enable unprecedented synthetic transformations.^22–30^ In recent years, transition metal-catalyzed C–H functionalization has been developed as a straightforward and efficient approach to constructing fused heterocyclic skeletons (Fig. 1).^25–50^ However, rhodium-catalyzed nondirected C–H activation/annulation to build fused heterocyclic cations has not been reported and remains a challenge since the regioselectivity is always an issue in the reactions with multiple analogous C–H bonds of (hetero)arenes. Here, we have deliberately designed substrates that enable straightforward and effective construction of pyrido-phenothiazin/phenoxazin/phenoselenazin/phenazin-12-iums *via* the rhodium-catalyzed nondirected C–H activation/annulation (Fig. 1).

## Results and discussion

We set out our investigation by using 10-methyl-10*H*-phenothiazine (1a) and 1,2-diphenylethyne (2a) as model substrates in the presence of [{RhCp*Cl_2_}_2_]/AgSbF_6_, Ag_2_O, and NaSbF_6_ in DCE at 120 °C for 48 hours, which afforded 2,3-diphenylpyrido[3,2,1-*kl*]phenothiazin-12-ium 3a in 44% yield (Table S1,† entry 1). After the optimization of the reaction conditions [see Tables S1 and S2 in the ESI†], we were able to improve the yield to 75% under reaction conditions comprising [{RhCp*Cl_2_}_2_] (5 mol%), AgSbF_6_ (40 mol%), Ag_2_O (1.5 equiv.) and NaSbF_6_ (1.5 equiv.) in DCE at 150 °C under N_2_ for 48 h (Table S1,† entry 12). It is noteworthy that this C–H activation/annulation reaction showed high regioselectivity at the methyl and C_1_ positions, and no C_3_-alkenylation product was detected.

Subsequently, a library of structurally diverse fused heterocyclic cations was synthesized using phenothiazines, phenoxazines, phenoselenazines, and phenazine compounds with alkynes under the optimized reaction conditions (Table 1, 3a–3n and 4a–4r). Diaryl alkynes with electron-neutral, electron-rich, and electron-deficient groups on the phenyl ring were successfully annulated and gave moderate to good yields of desired products (3a–3m). Dialkyl acetylene was also annulated with 10-methyl-10*H*-phenothiazine and provided 3n in 52% yield (Table 1). Unsymmetrical alkynes were used to annulate with 10-methyl-10*H*-phenothiazine, giving moderate yields (Table 1, 3o–3r). The PhC≡CH and 1-(phenylethynyl)-4-(trifluoromethyl)benzene reacted with compound 1a to give two regioisomeric products with a ratio of 1 : 1 (Table 1, 3q–3r). To our delight, prop-1-yn-1-ylbenzene and methyl 3-phenylpropiolate underwent annulation with 1a to generate products 3o and 3p with excellent regioselectivity (Table 1, Fig. S20 and 21†). It should be mentioned that various functional groups such as fluorides, chloride, bromide, phenoxy, cyano, and carboxylic ester were well tolerated under the reaction conditions (3f–3j, 4h, 4j–4n), which provides an ample opportunity for further manipulation of the initial products. Derivatives of dicyanoisophorone (DCI) tend to possess ultra-fast intramolecular charge transfer and were used as fluorescence probes with large Stokes shifts. Notably, the DCI unit was well tolerated in the reaction, and the corresponding product was isolated in moderate yield (4o). Furthermore, the structure of 4d was confirmed by single-crystal X-ray analysis (Fig. S18†).

**Table 1:** Rhodium-catalyzed construction of fused heterocyclic cations^a^

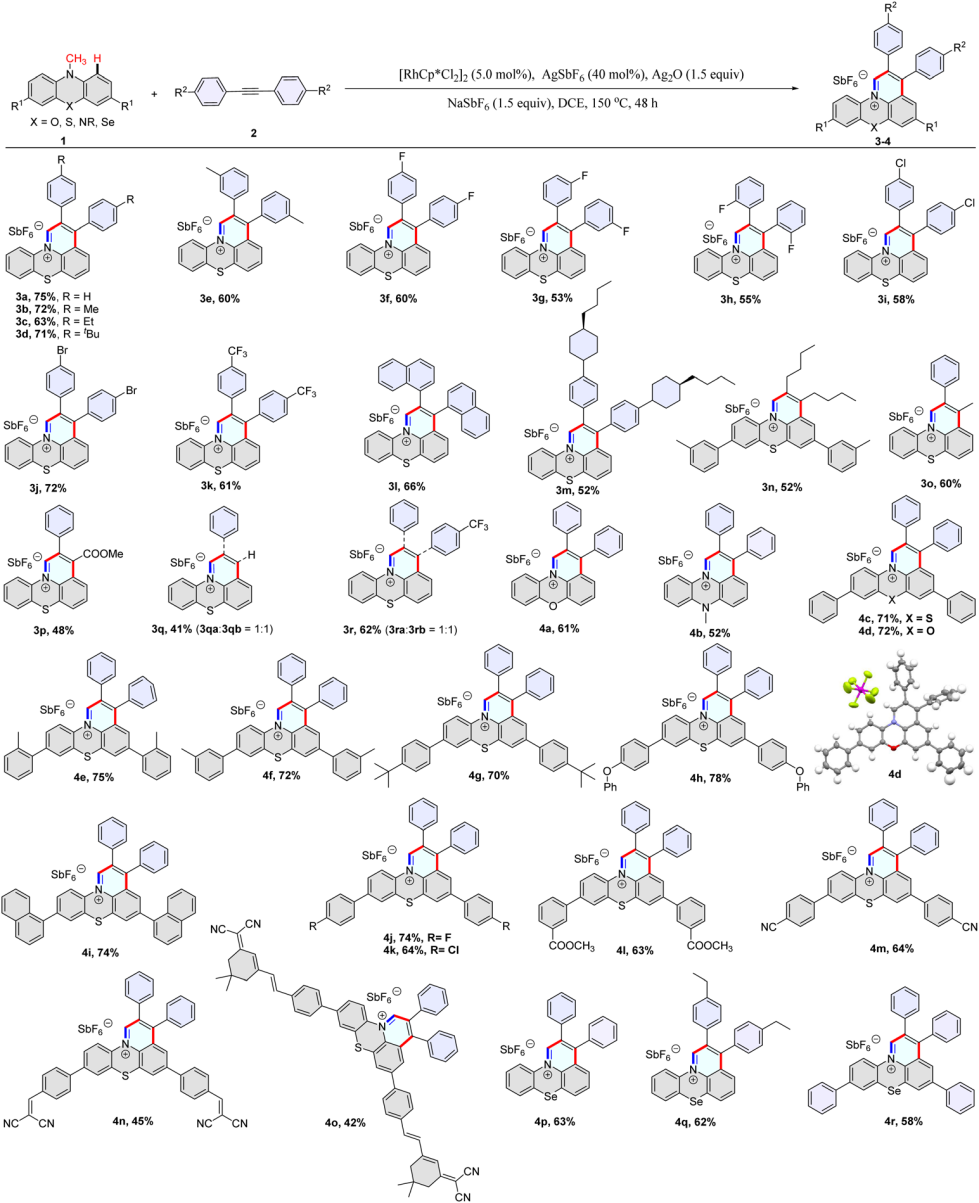

a Reaction conditions: 1 (0.1 mmol), 2 (0.15 mmol), [Cp*RhCl_2_]_2_ (5.0 mol%), AgSbF_6_ (40 mol%), Ag_2_O (1.5 equiv.), NaSbF_6_ (1.5 equiv.), DCE (1 mL), at 150 °C, 48 h under a N_2_ atmosphere.

To gain mechanistic insight into the C–H activation/annulation process, a series of control experiments were performed. In the H/D exchange experiment, 10% and 67% of deuterium incorporation were found at the C_1_ and C_3_-positions of 10-methyl-10*H*-phenothiazine (1a) in the presence of CD_3_OD under varied reaction times (Scheme S2 and Fig. S1†), whereas there is no H/D exchange at the methyl position of 10-methyl-10*H*-phenothiazine (1a). These results indicated that the C–H bond activation step might be reversible, the C–H bonds at the C_1_ and C_3_-positions of 1a have similar reactivity, and the reaction might begin with the metalation of the C_1_ or C_3_-position. Subsequently, the kinetic isotope effect (KIE) was investigated, and two different KIE values were obtained (Schemes S3 and S4, and Section S5 of the ESI†). A significant KIE value (*k*_H_/*k*_D_ = 2.4) between 1a and [D]_3_-1a with 2a was observed, suggesting that the C(sp^3^)–H bond cleavage of the methyl group of 1a might be involved in the rate-limiting step. Furthermore, the radical scavenger 2,2,6,6-tetramethyl-1-piperidinyloxy (TEMPO) was added to the reaction and showed a negligible effect, which ruled out a radical pathway (Table S1,† entry 18).

**Fig. 2:**
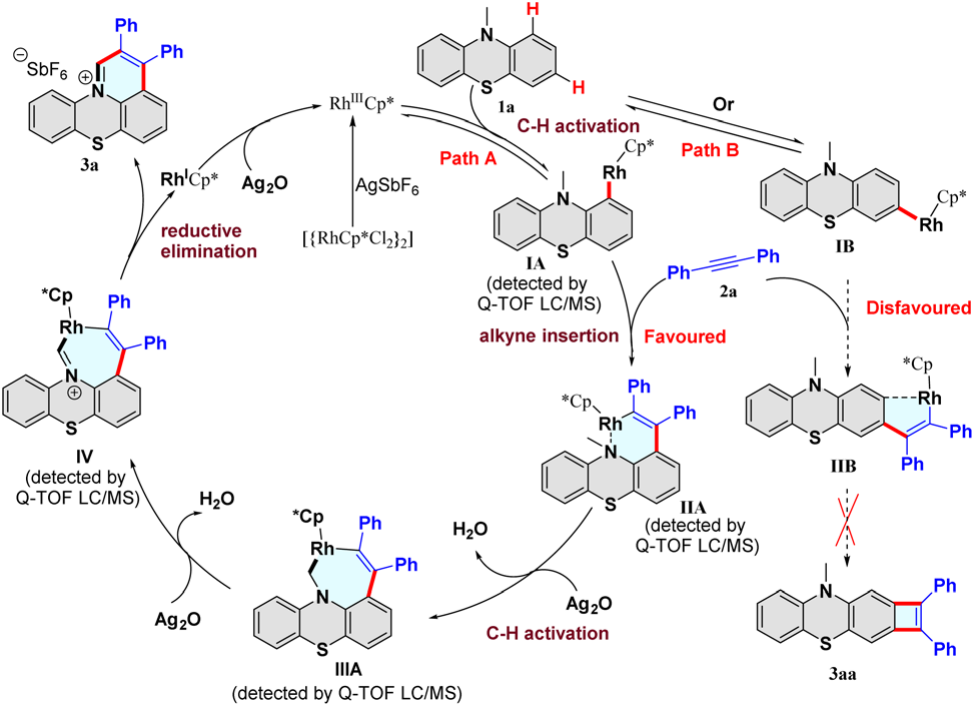
Proposed mechanism.

Based on these results and previous reports,^35–50^ a plausible catalytic cycle is proposed (Fig. 2). First, catalyst [Cp*RhCl_2_]_2_ reacts with AgSbF_6_ to offer the highly electrophilic [Rh^III^Cp*] species. In path A, the substrate 1a reacts with [Rh^III^Cp*] to form the intermediate IA after a reversible C–H bond cleavage process at the C_1_-position. Next, the alkyne 2a coordinates with the rhodium intermediate IA, and subsequently inserts into the Rh–C bond to provide the intermediate IIA. Then, the intermediate IIA undergoes rhodium-catalyzed C(sp^3^)–H bond activation to form the seven-membered rhodacycle intermediate IIIA. Finally, dehydrogenation of IIIA afforded the intermediate IV, followed by a reductive elimination process to release the desired product 3a. The formed rhodium(i) species is oxidized to the [Rh^III^Cp*] by Ag_2_O. Furthermore, the generation of all the intermediates IA–IV was supported by high-resolution electrospray ionization mass spectrometry (HRMS) analysis (Fig. S3–S6†). In path B, substrate 1a reacts with [Rh^III^Cp*] to generate the intermediate IB after a reversible C–H bond cleavage process at the C_3_-position. Then, alkyne inserts into the IB to form the intermediate IIB. However, forming the rigid four-membered product 3aa is difficult due to its thermodynamic instability. Notably, we did not observe the four-membered compound 3aa in the reaction. Consequently, path B is disfavored and could be excluded from the reaction. Hence, the C–H activation/annulation reaction showed high regioselectivity at the methyl and C_1_ positions, and no C_3_-position product was detected.

Organic fluorescence materials, especially single-molecule-white-light emitting materials, and red-light emitting materials, have attracted considerable attention in recent years due to their applications in organic light-emitting diodes, field-effect transistors, security systems, fluorescent markers, bioprobes, *etc.*^51–61^ However, discovering single-molecule white-light materials is a challenging task because white-light-emission involves a broad emission covering the whole visible range (400–700 nm), which is difficult for ordinary organic fluorescent molecules due to their intrinsic limitation of photophysical properties. Therefore, organic single-molecule white-light materials are scarce, and only a few have been developed so far. Compounds with anti-Kasha systems have great potential for preparing single-molecular white-light-emitting materials because of their double emissions with a relatively short wavelength (blue-light component) from a high-lying excited state emission (S_*n*_ → S_0_, *n* ≥ 2), and a relatively long wavelength (orange-light component) from a low-lying excited state emission (S_1_ → S_0_).^56–58^

**Fig. 3:**
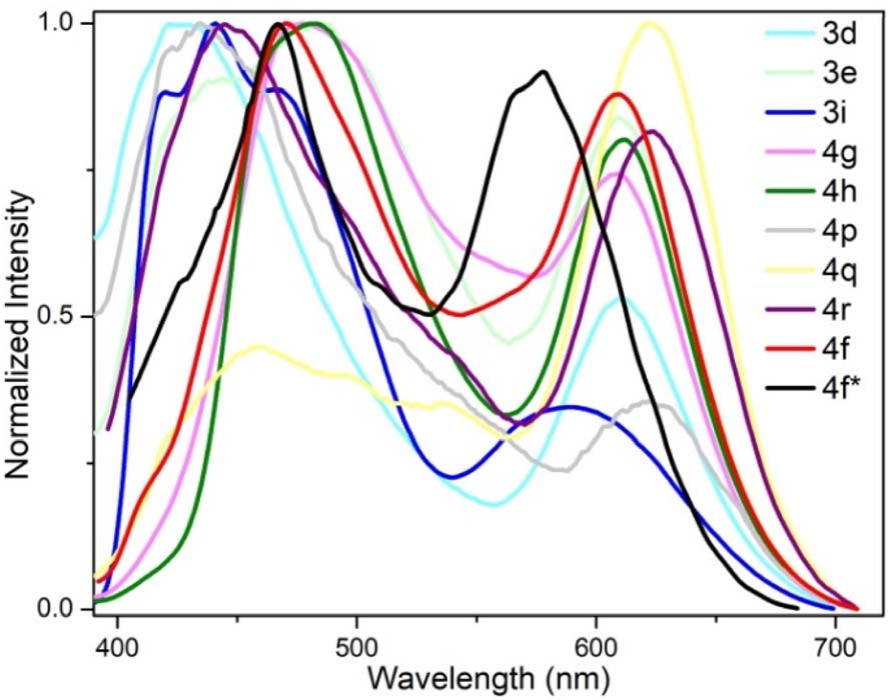
Fluorescence properties. Fluorescence emission spectra of 3d, 3e, 3i, 4f–4h, 4p–4r in CH_2_Cl_2_ and 4f* in the PMMA film.

**Fig. 4:**
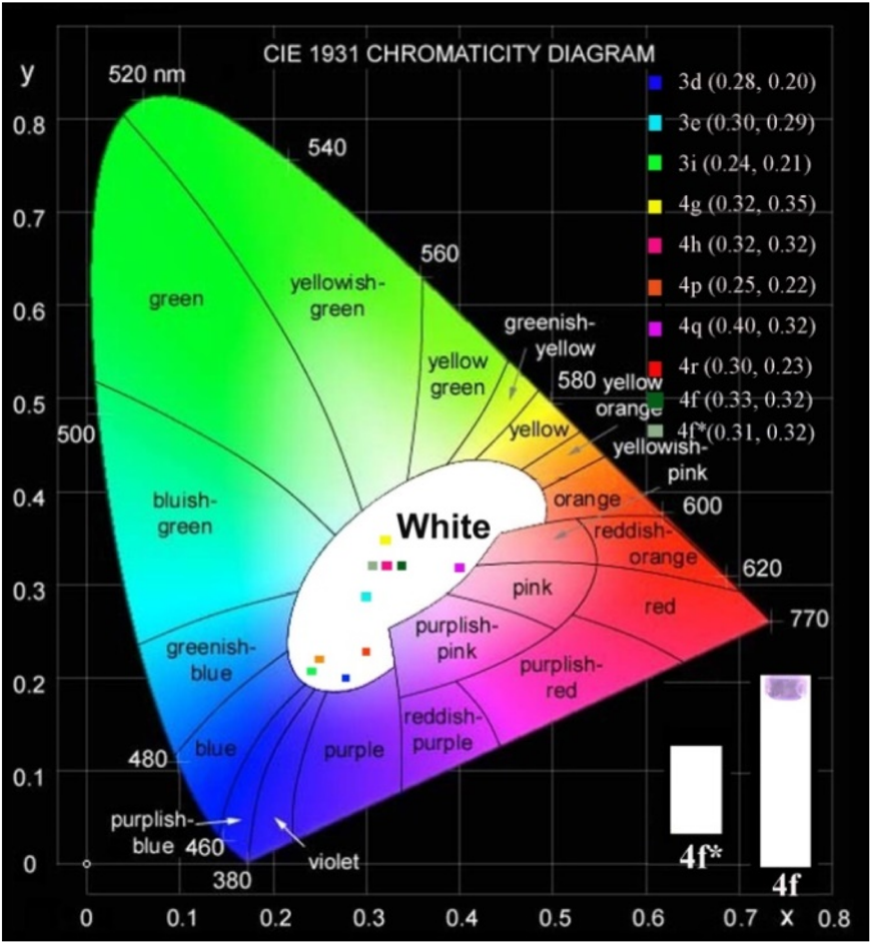
Chromatic coordinates. Commission Internationale de l'Eclairage (CIE) coordinates of 3d (0.28, 0.20), 3e (0.30, 0.29), 3i (0.24, 0.21), 4g (0.32, 0.35), 4h (0.32, 0.32), 4p (0.25, 0.22), 4q (0.40, 0.32), and 4r (0.30, 0.23) in CH_2_Cl_2_, respectively.

**Fig. 5:**
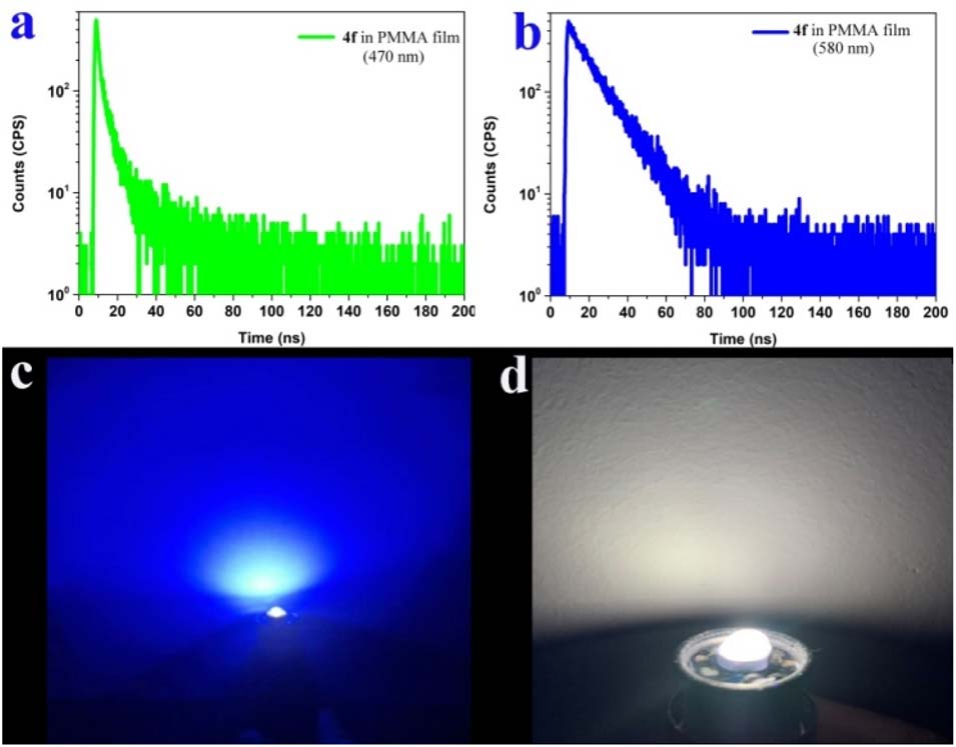
Transient emission spectra and luminescence image. (a) and (b) Transient emission spectra of 4f in the PMMA film at room temperature. (c) Luminescence image of a commercially available UV lamp. (d) Luminescence image of a UV chip coated with the 4f film (0.005 wt% in PMMA) when the LED is turned on.

We further measured the photophysical properties of the constructed library of pyrido-phenothiazin/phenoxazin/phenoselenazin/phenazin-12-iums, and the corresponding absorption and emission maxima are summarized in Table S3 and Fig. S8–11.† To our delight, these compounds exhibited distinct dual emissions with a relatively short blue emission (anti-Kasha emission) and a relatively long orange emission wavelength (Kasha emission), which thus efficiently covers the whole visible range (400–700 nm). Fortunately, nine fused heterocyclic cations 3d, 3e, 3i, 4f, 4g, 4h, 4p, 4q, and 4r displayed white light emissions in dichloromethane with Commission Internationale de l'Eclairage (CIE) coordinates of 3d (0.28, 0.20), 3e (0.30, 0.29), 3i (0.24, 0.21), 4f (0.33, 0.32), 4g (0.32, 0.35), 4h (0.32, 0.32), 4p (0.25, 0.22), 4q (0.40, 0.32), and 4r (0.30, 0.23), respectively (Fig. 3, 4 and S12†). Compound 4f displayed dual emission with blue emission at approximately 470 nm and orange emission at 610 nm with a fluorescence quantum yield of 10% in dichloromethane (Fig. 3). It is worth pointing out that 4f emits bright white light with CIE coordinates of (0.33, 0.32), very close to those of pure white light (CIE: 0.33, 0.33). Moreover, 4f exhibits white-light emission in polymethyl methacrylate (PMMA) film (*c* = 0.005 wt%) with CIE coordinates of (0.31, 0.32) and a fluorescence quantum yield of 20% (Fig. 3 and 4). In addition, the excited-state lifetimes of 4f in the dichloromethane solution and in the PMMA film were tested at room temperature (Table S4 and Fig. S14†). It is observed that the radiative excitons in 4f are short-lived components with nanosecond order (Fig. 5a and b), and no long-lifetime fluorescence components exist, which ruled out the delayed fluorescence or phosphorescence emission. The thermogravimetric analyzer measurement demonstrates that 4f is thermally stable (Fig. S15†).

White light-emitting diodes (LEDs) have received much attention and have been widely used in solid-state lighting displays and illumination.^61–63^ Currently, most commercial white light-emitting diodes are made by coating green or inorganic phosphors on LED chips.^61–63^ To further prove the potential application of our white-light materials, we coated the 4f film (0.005 wt% in PMMA) on a commercially available UV chip, and bright white light could be obtained when the LED is turned on (Fig. 5c and d). This work unlocks an opportunity to rapidly fabricate robust organic and low-cost white LEDs.

**Fig. 6:**
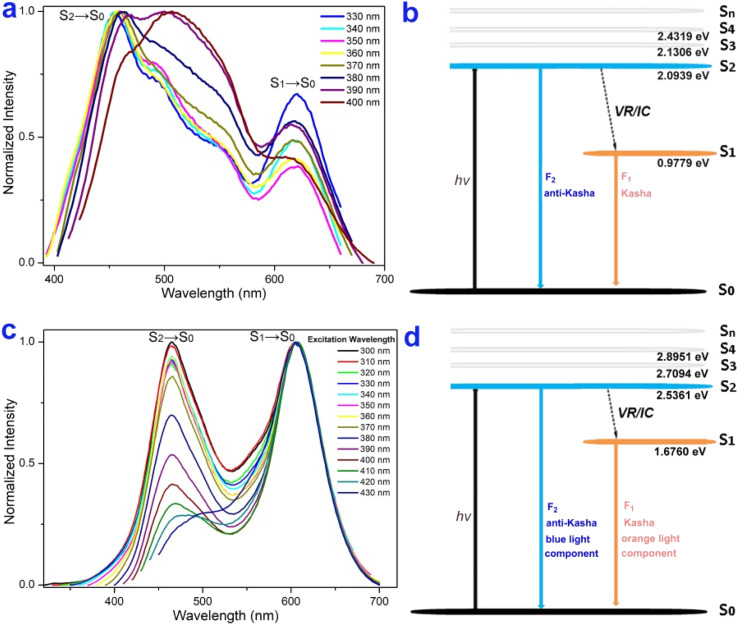
Anti-Kasha dual-emission character. (a), and (c) Excitation-wavelength-dependent fluorescence spectra of 4p and 4f (1.0 × 10^−6^ M). (b), and (d) Jablonski diagram illustrating the anti-Kasha dual-emission mechanism of 4p and 4f.

The excitation-wavelength-dependent fluorescence experiments and theoretical calculations further demonstrated that 4p and 4f possess an anti-Kasha dual-emission character.^56–58,64,65^ The relative intensity of dual emission highly depends on the excitation wavelength. For 4p, lower energy excitations result in a slight red-shift at shorter wavelengths, and the intensity of longer wavelengths decreases and then increases (Fig. 6a). For 4f, higher energy excitations resulted in enhanced emissions at shorter wavelengths (Fig. 6c). This experiment implied that the two fluorescence bands were from different excited states: the white light emission of 4f with the blue-light component from a high-lying excited state emission (S_2_ → S_0_), and the orange-light component from a low-lying excited state emission (S_1_ → S_0_) (Fig. 6c). The internal conversion (IC) from the S_2_ to the S_1_ state is comparatively slow due to the large energy gap Δ*E*(S_2_ → S_1_) value (4p: 1.116 eV, 4f: 0.6633 eV), and as a result, S_2_ fluorescence can compete favorably with IC (Fig. 6b and d).

**Fig. 7:**
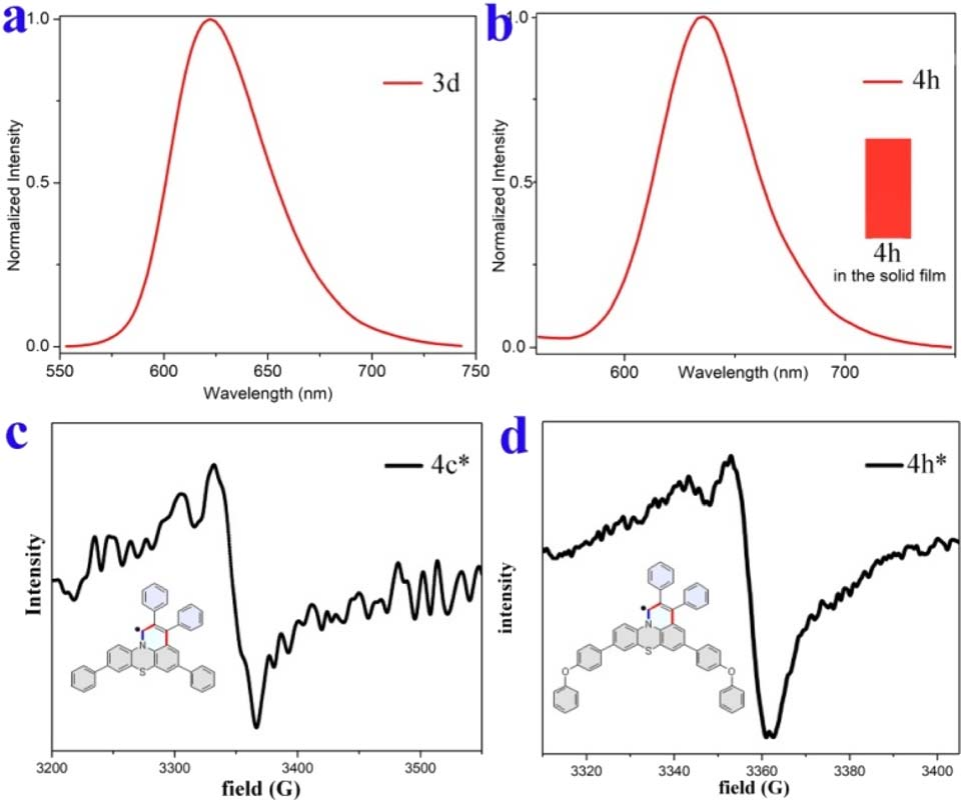
Fluorescence spectra and EPR spectra. (a) Fluorescence emission spectrum of 3d in the solid film. (b) Fluorescence image and emission spectrum of 4h in the solid film. (c) and (d) EPR spectra of reduced 4c and 4h in the solid state at room temperature.

Furthermore, compounds 3d and 4h in solid films displayed only low energy emission (Kasha emission) due to strong π–π interactions. Compound 3d showed red-light emission at 622 nm with CIE coordinates of (0.66, 0.33) (Fig. 7a and S13†). The 4h exhibited pure red-light emission at 636 nm with a fluorescence quantum yield of 5% and CIE coordinates of (0.67, 0.33) (Fig. 7b), which is consistent with the National Television System Committee (NTSC) standard red CIE coordinates of (0.67, 0.33). The thermogravimetric analyzer measurement showed that 4h is thermally stable (Fig. S15†).

π-conjugated radicals have recently attracted immense attention because of their excellent optical, electronic, and unique magnetic properties, and thus have a range of potential applications in nonlinear optics, spintronics, organic electronics, and energy storage devices.^66–68^ Herein, our newly developed fused heterocyclic cations are easy to convert into stable π-conjugated radicals. As illustrative examples, the cations 4c and 4h could be easily reduced to π-conjugated radicals by using NaI in an acetonitrile solution (see Section IX of the ESI†). The corresponding reduced products are electron paramagnetic resonance (EPR) active and offer *g* values of 2.0025 and 2.0023, respectively, which are almost equal to the *g* factor for a free electron that of the typical organic radicals (*g* = 2.0023) (Fig. 7c and d).

## Conclusions

In conclusion, rhodium-catalyzed nondirected C–H activation/annulation has been developed to build a library of structurally diverse pyrido-phenothiazin/phenoxazin/phenoselenazin/phenazin-12-iums for the first time. The protocol shows excellent regioselectivity and broad substrate scope. Deuterium-labeling experiments have indicated that the C(sp^3^)–H bond cleavage of the *N*-methyl group might be involved in the rate-limiting step in the catalytic process. Furthermore, this approach provides straightforward access to highly π-conjugated fused heterocyclic cations, which opens up a new avenue for rapid screening of single-molecular white-light-emitting and pure red-light-emitting materials. More importantly, novel π-conjugated fused heterocyclic cations displayed anti-Kasha doublet emissions and were used as an excellent white light source to rapidly fabricate robust organic and low-cost white LEDs. Additionally, these π-conjugated fused heterocyclic cations could readily be converted into stable π-conjugated radical materials. The highly efficient gateway toward single-molecular white-light-emitting and pure red-light emitting materials and π-conjugated radical materials developed herein has highlighted the exceptional charm of C–H functionalization for streamlining the lead-optimization phase in the discovery of organic functional materials.

## Data availability

All experimental data associated with this work are provided in the ESI.†

## Author contributions

J. Z., T. S., K. W., R. H. and C. Z. performed the experiments and analyzed the data. H. G. and B. L. designed and directed the project and wrote the manuscript. All authors contributed to discussions.

## Conflicts of interest

There are no conflicts to declare.

## Supplementary Material

SC-015-D4SC02188F-s001

SC-015-D4SC02188F-s002
