# Supplementary figures and images for: Rh(iii)-catalyzed building up of used heterocyclic cations: facile access to white-light-emitting materials
Source: Chem Sci. 2024 Jun 11;15(31):12270–6. doi: 10.1039/d4sc02188f (PMC11304525; doi:10.1039/d4sc02188f)

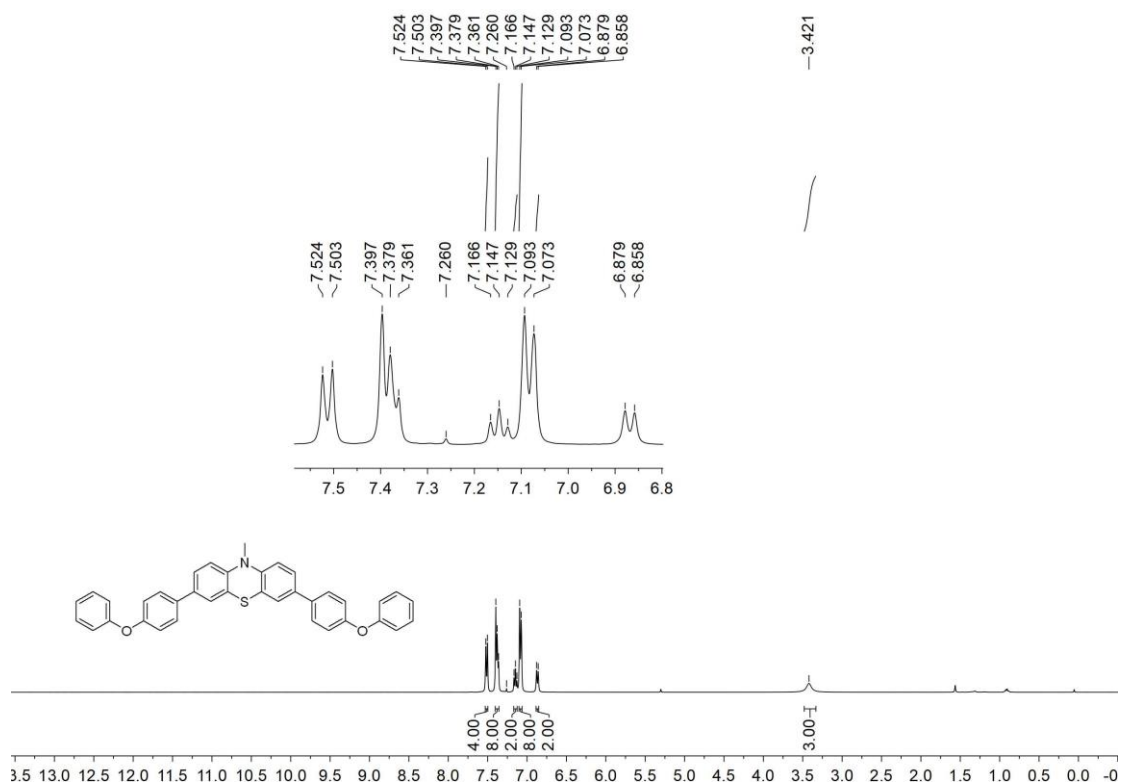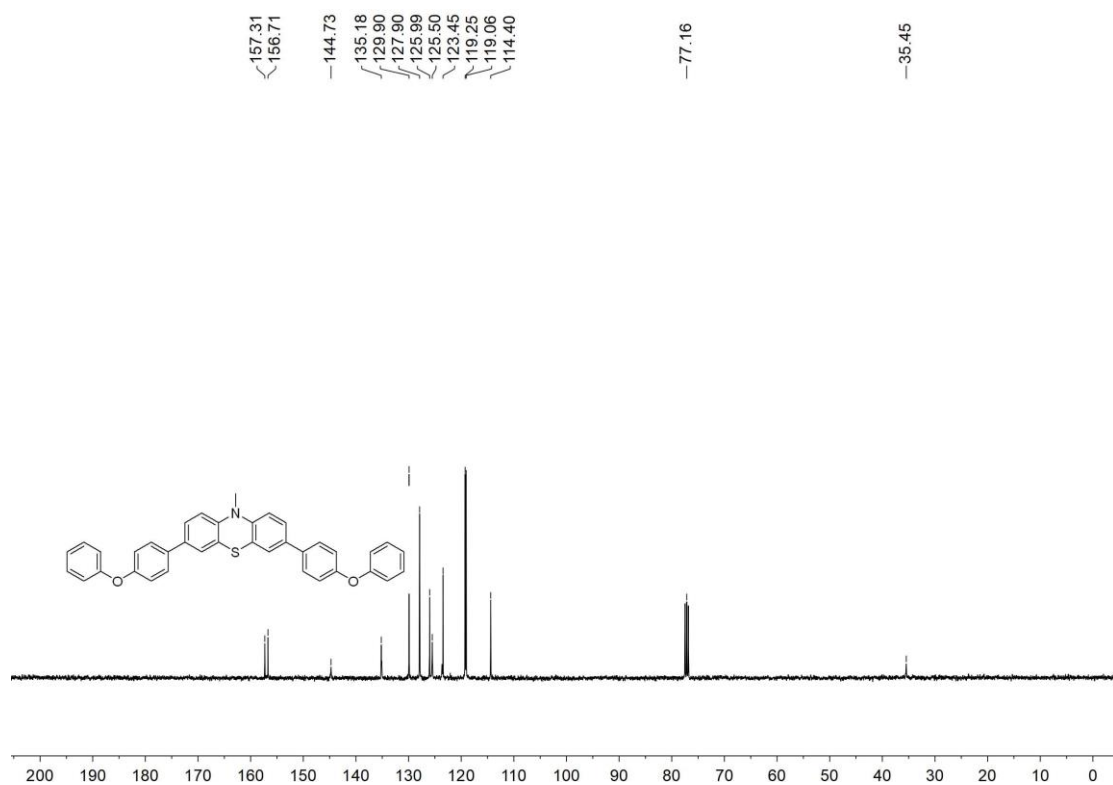



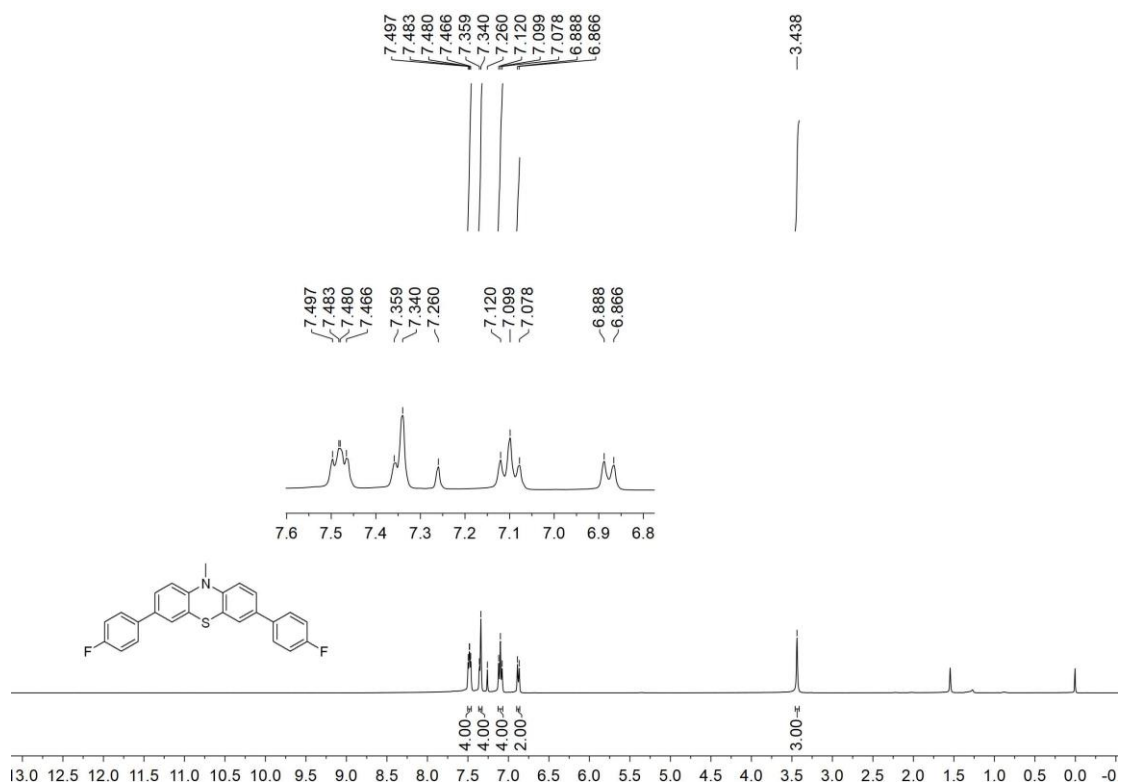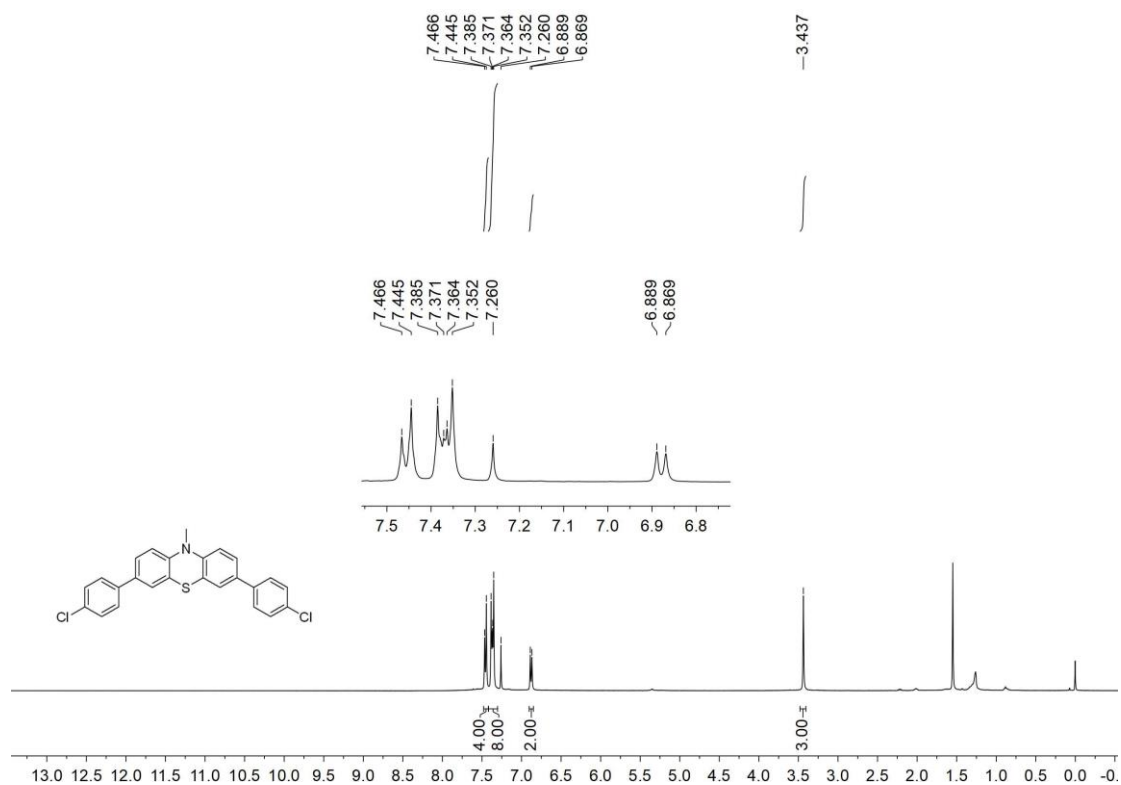

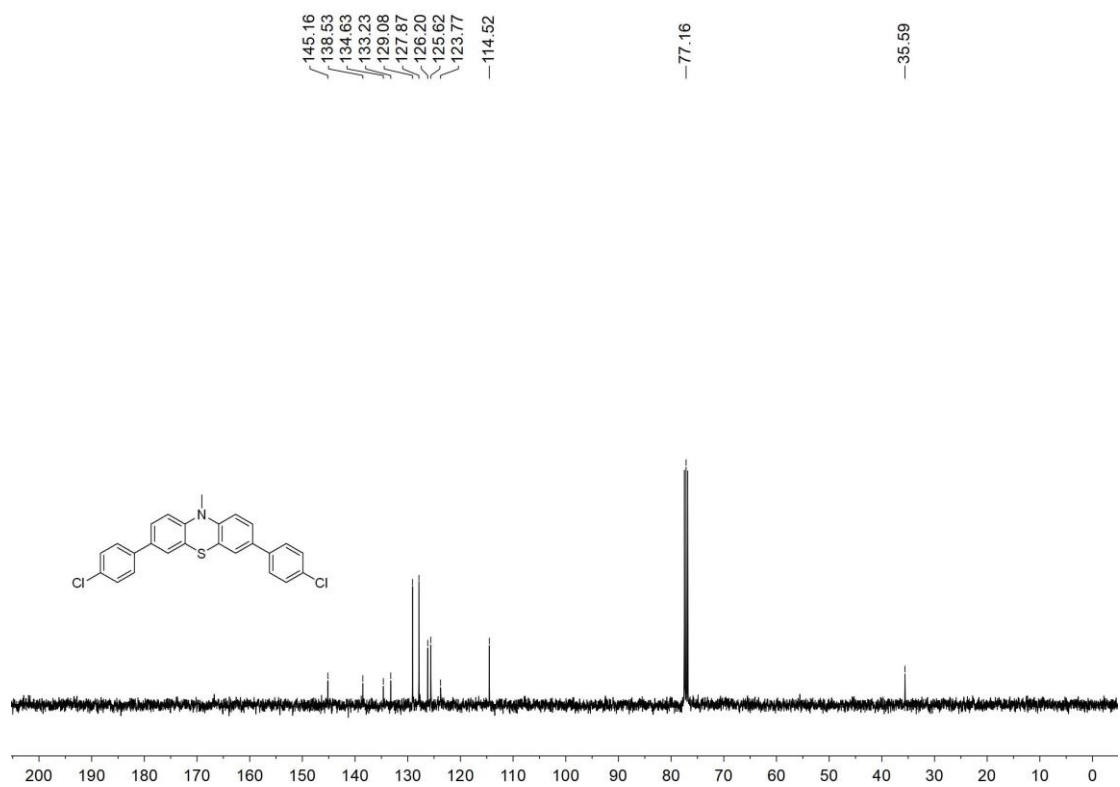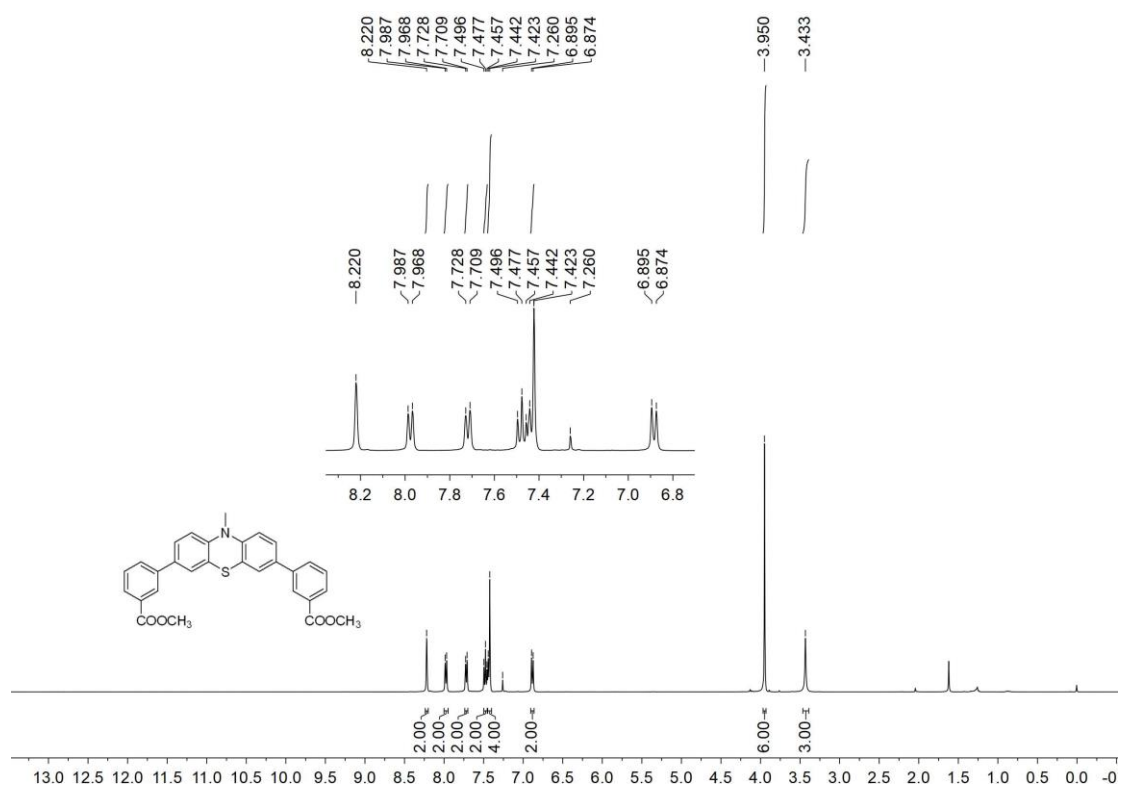

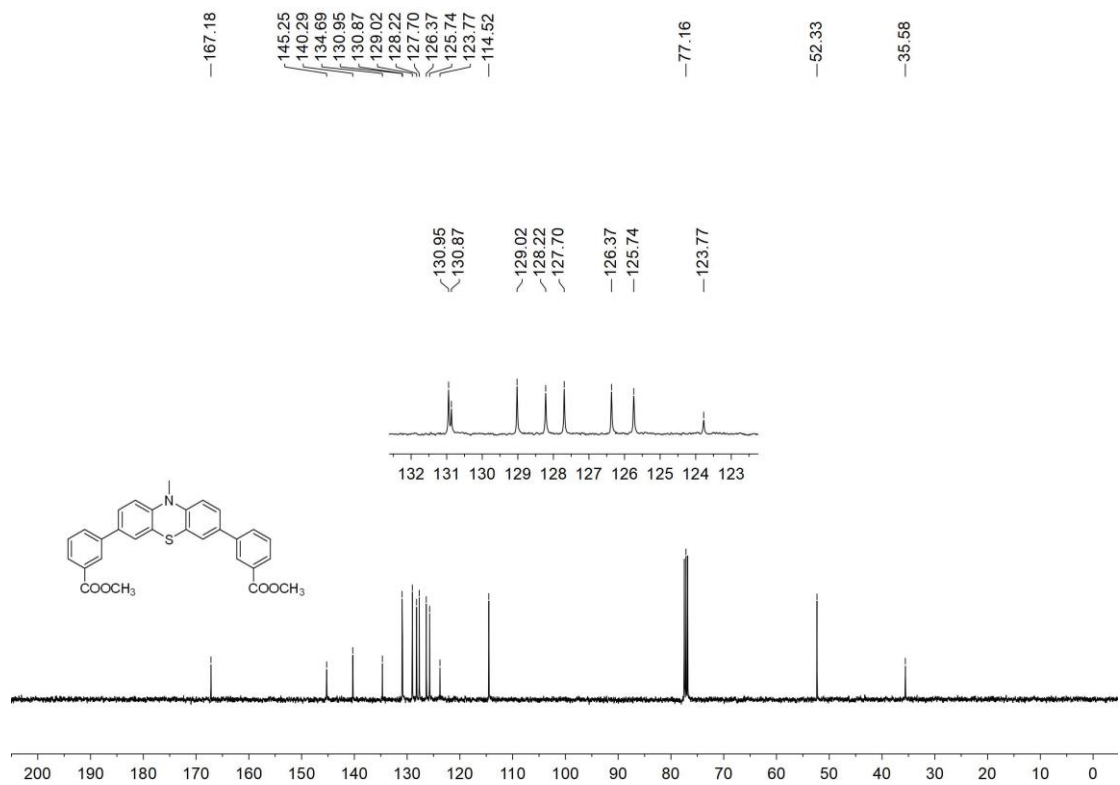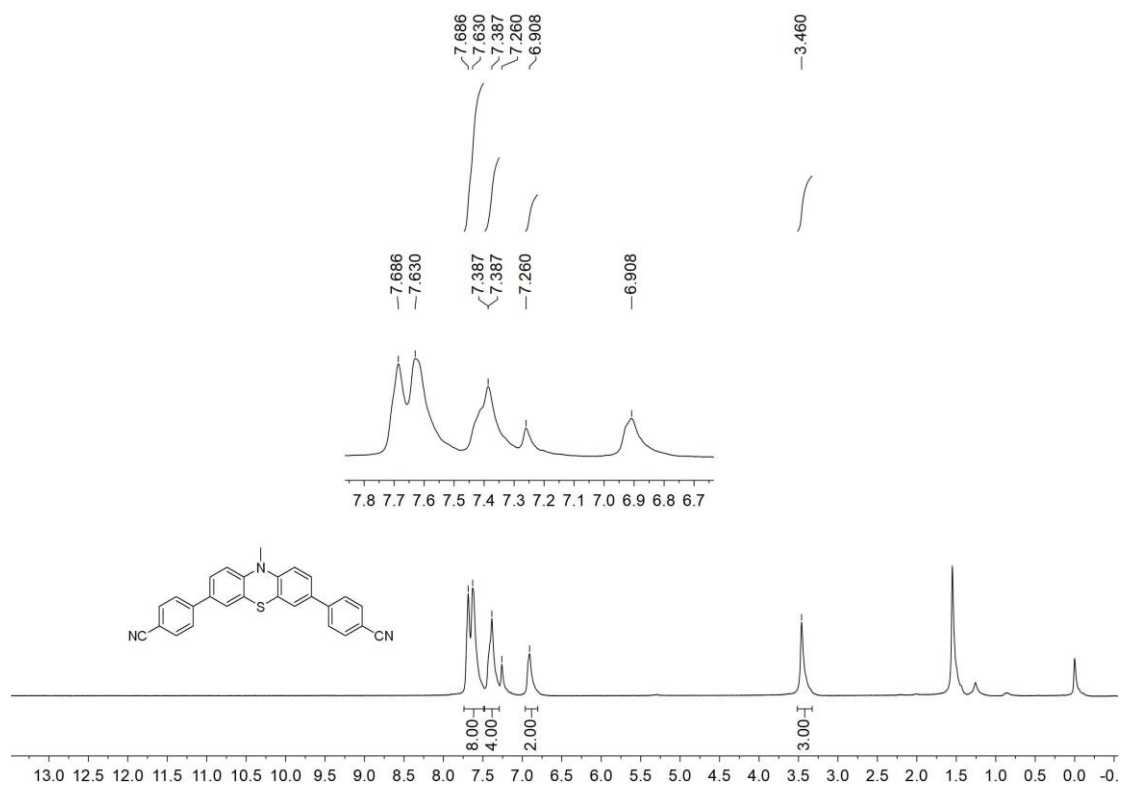

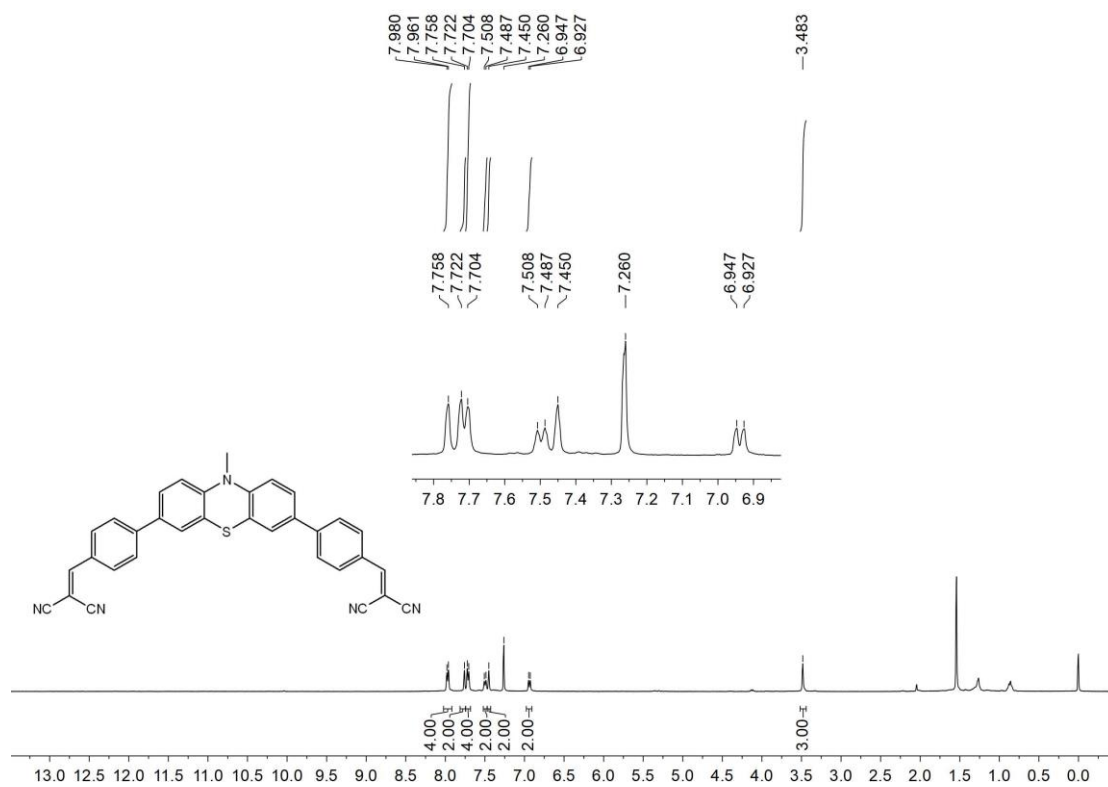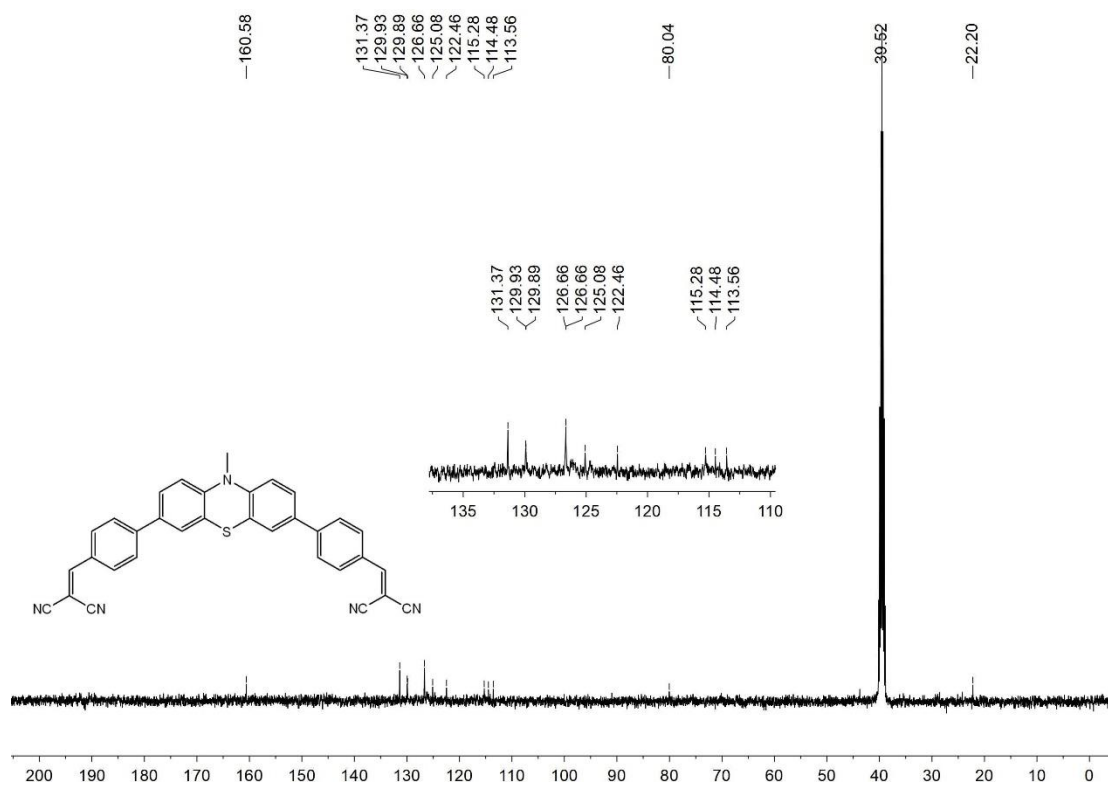

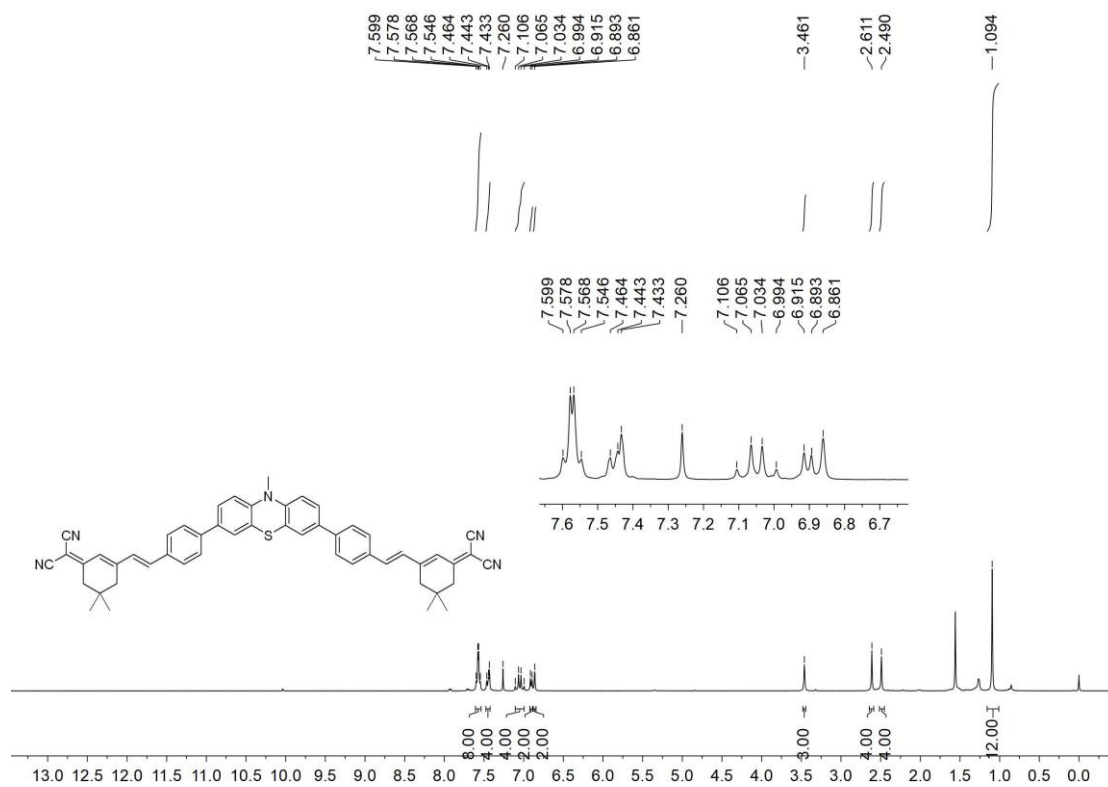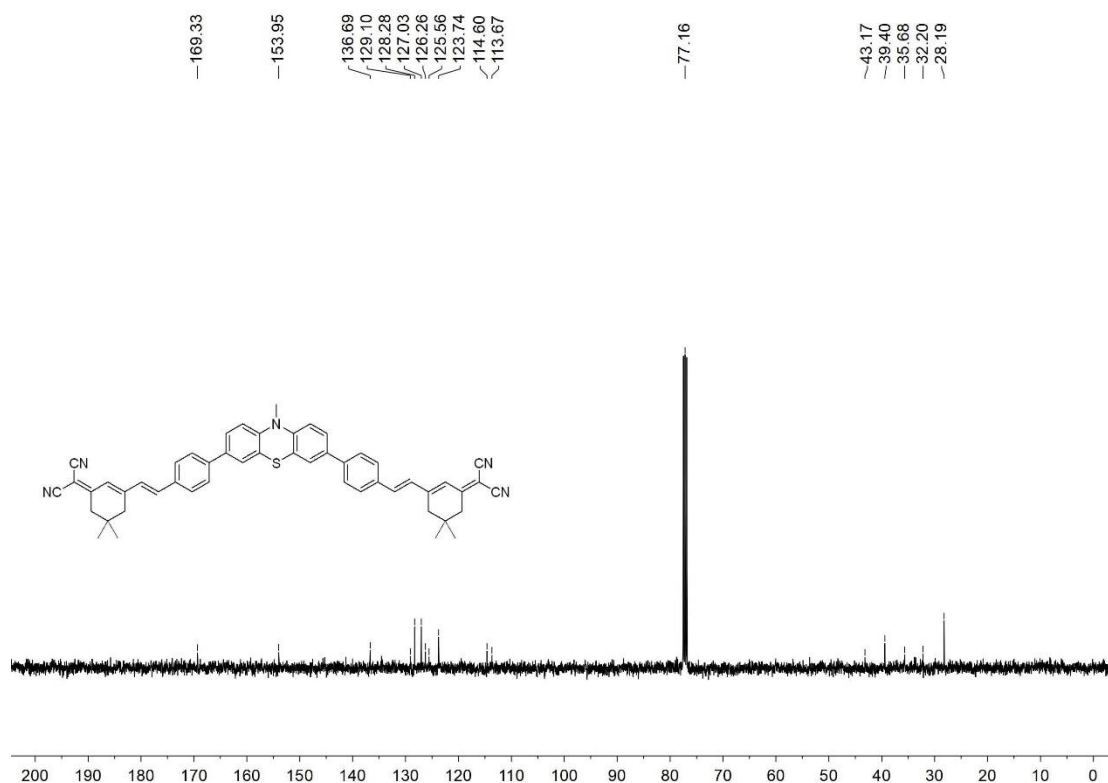

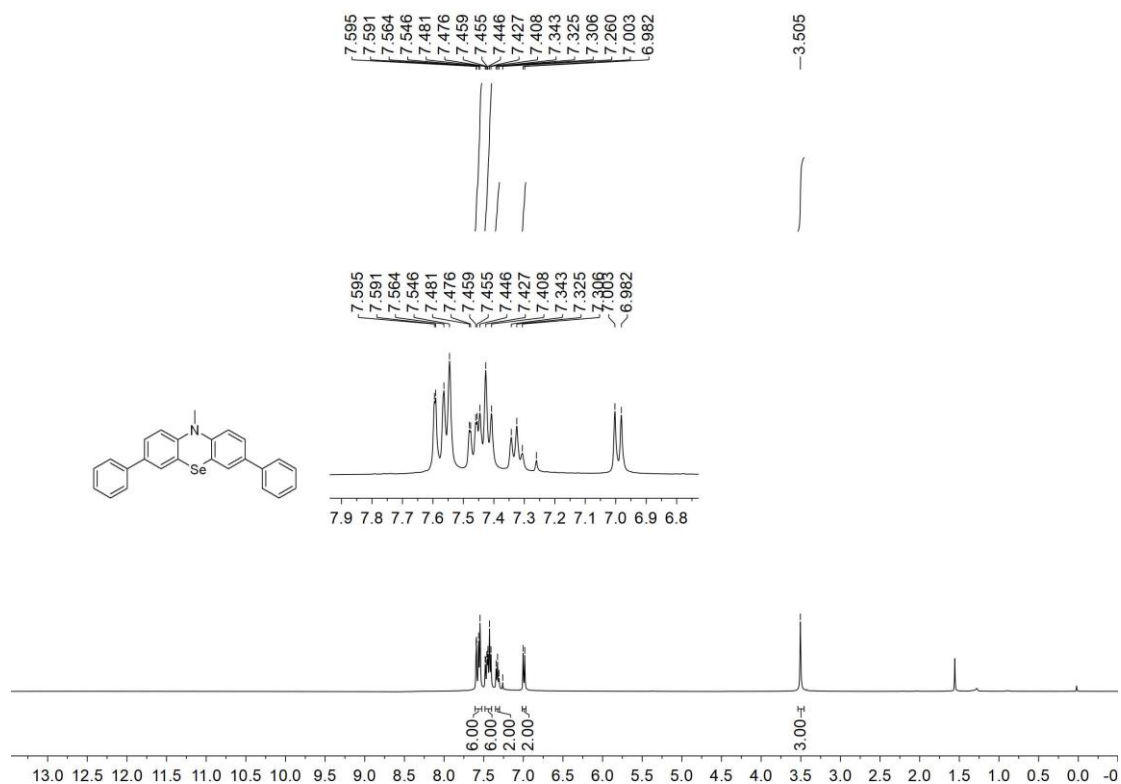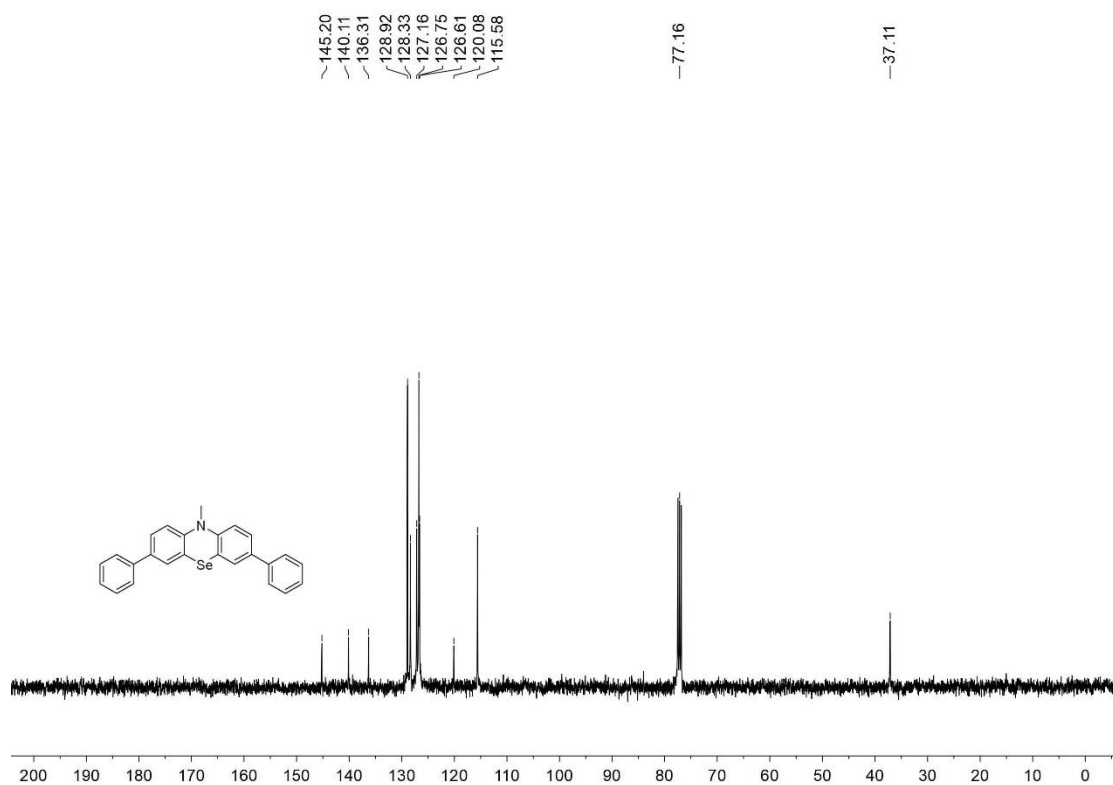

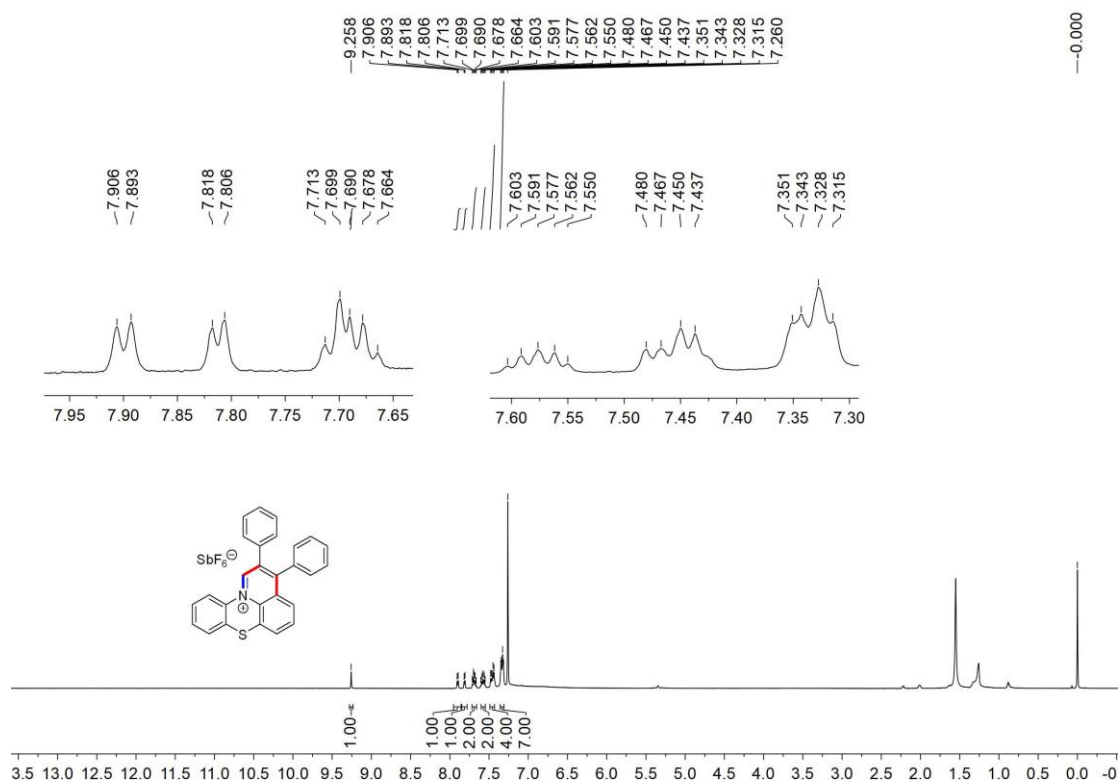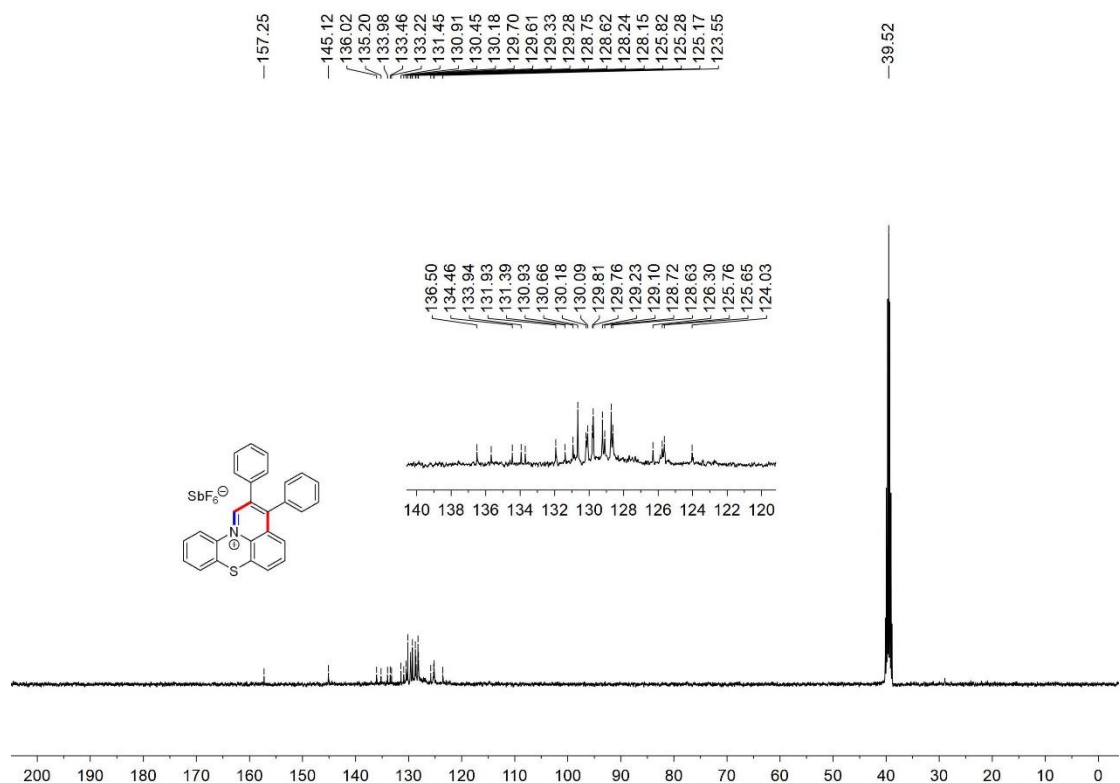

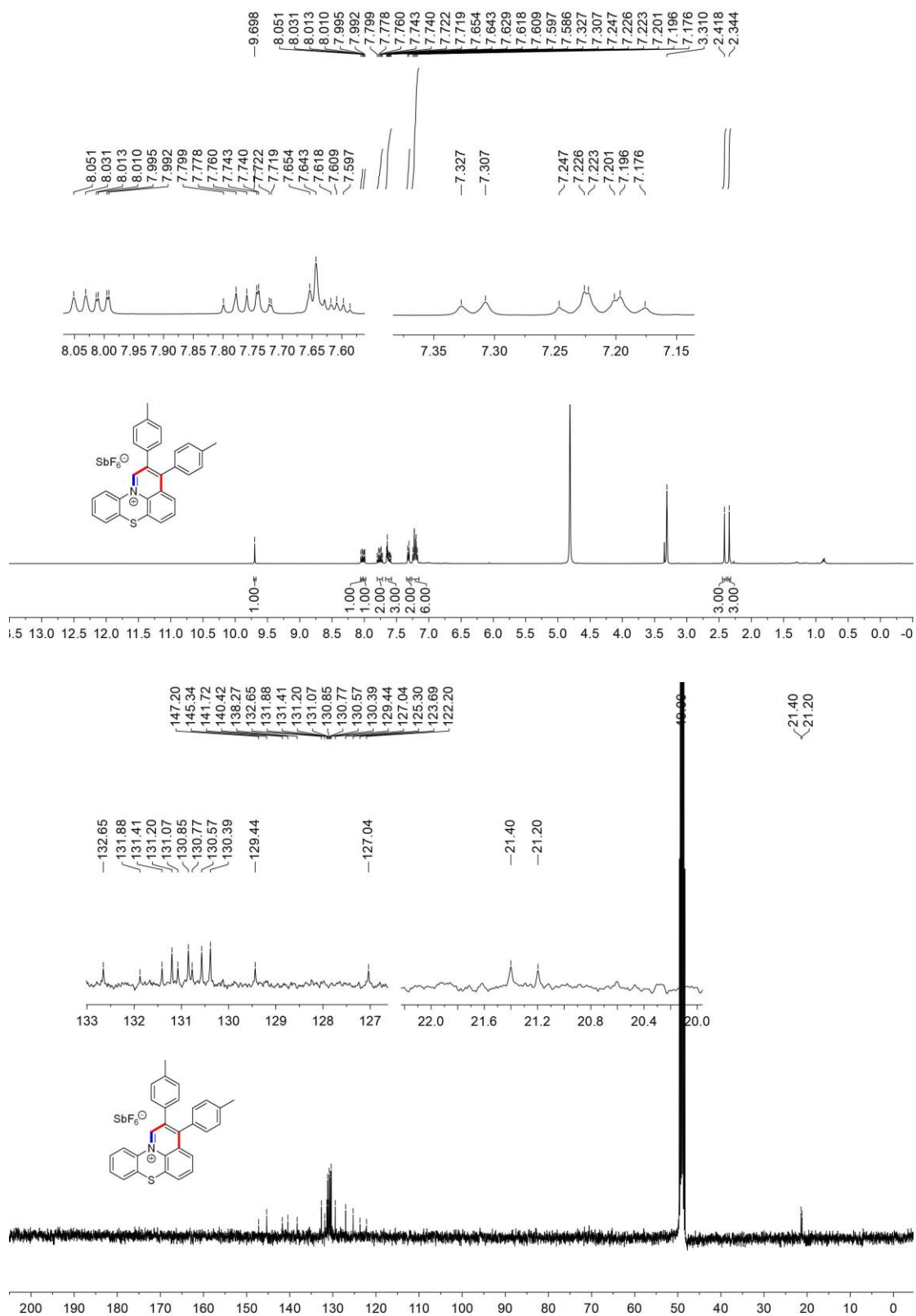

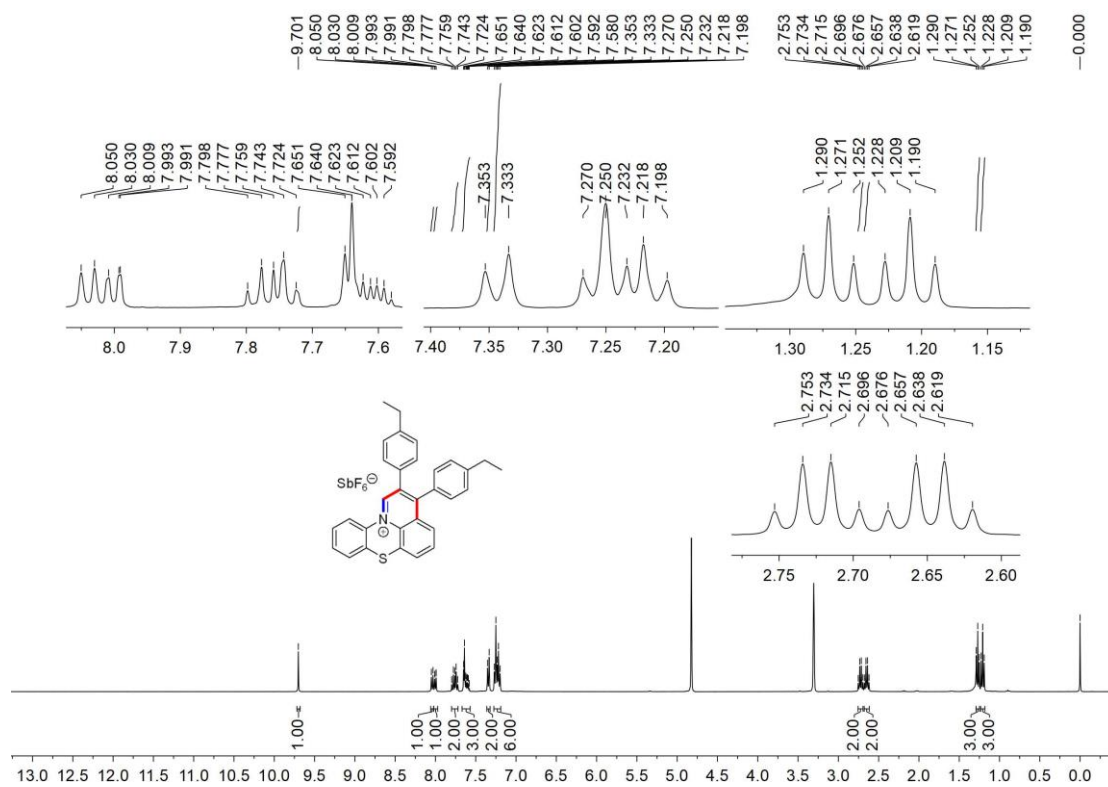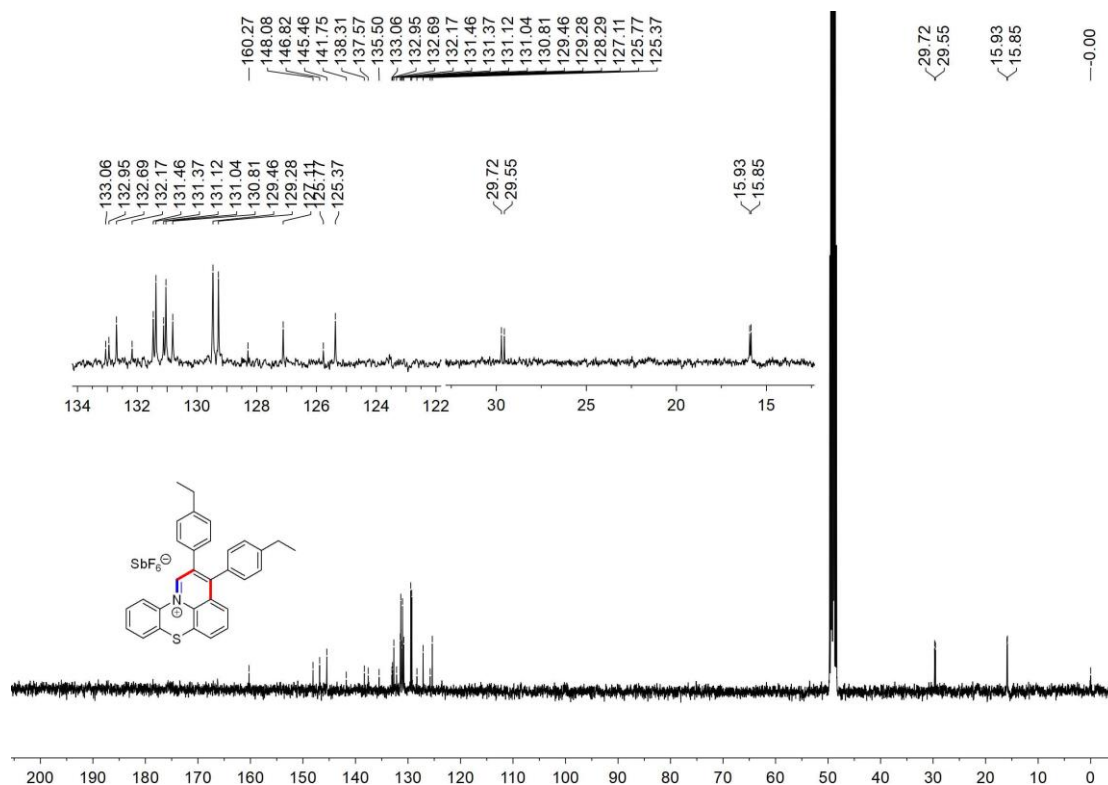

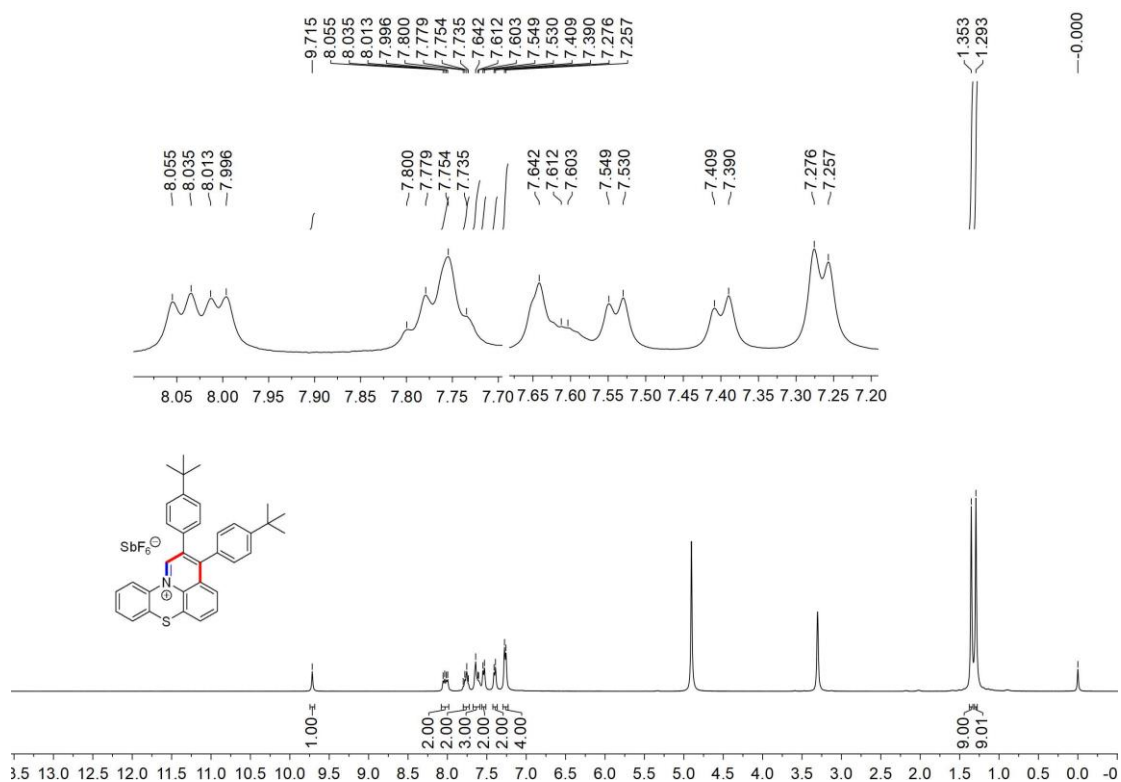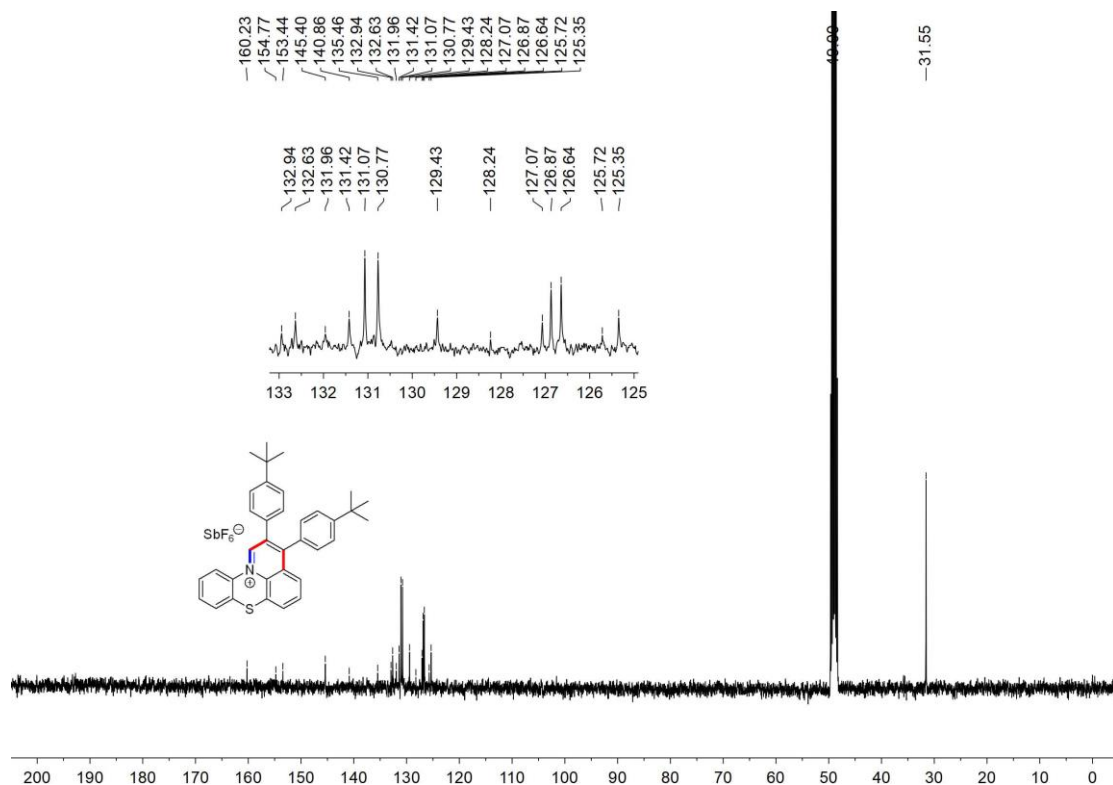

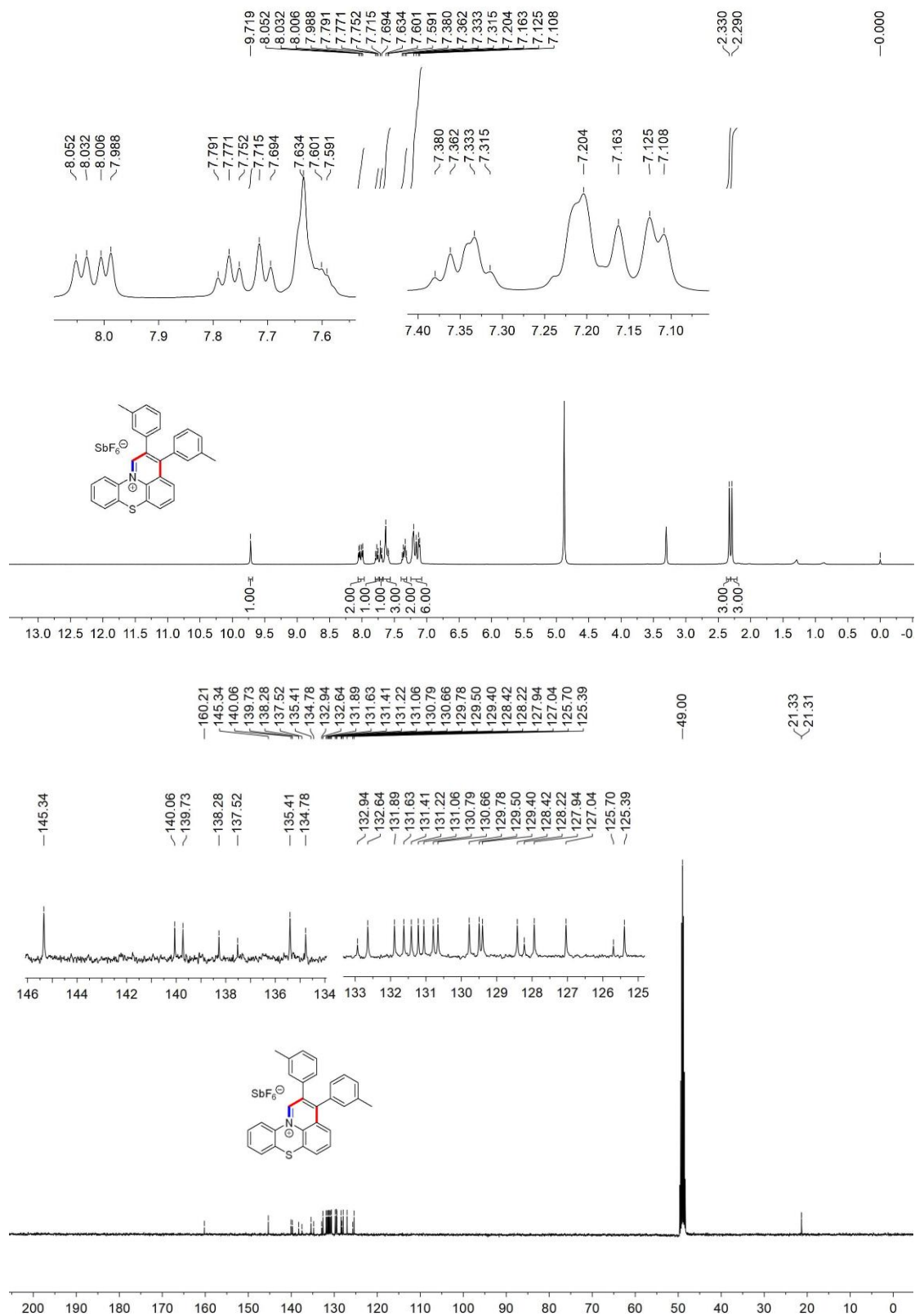

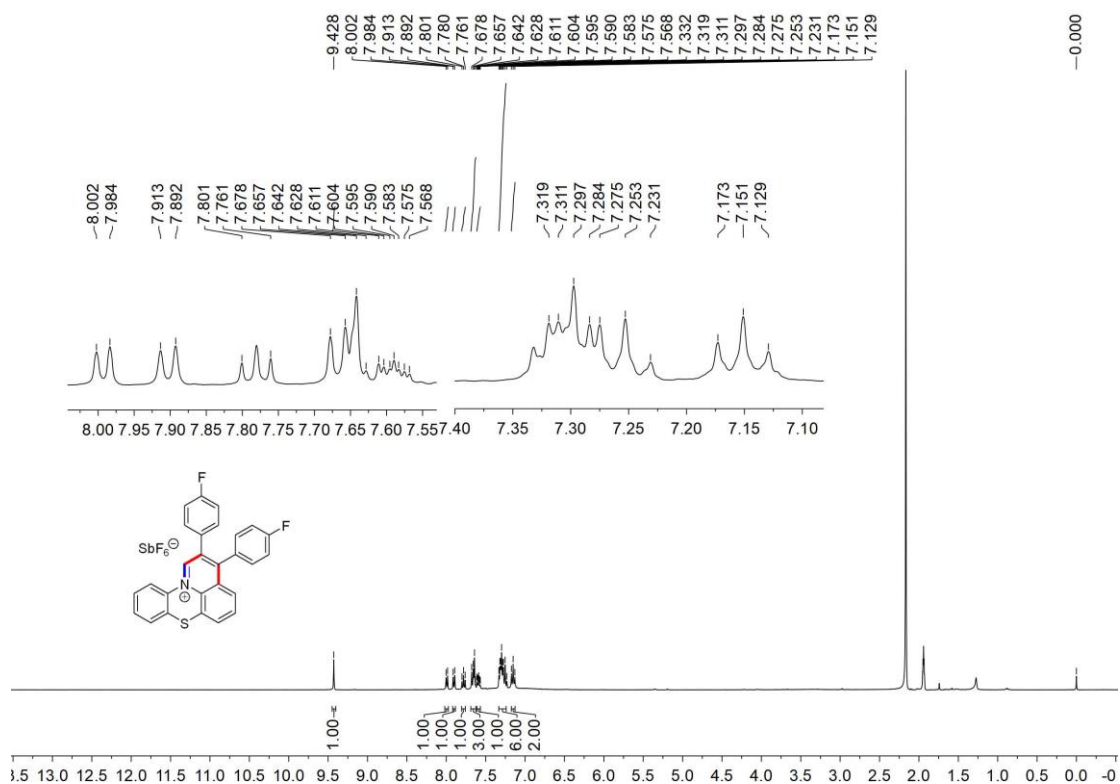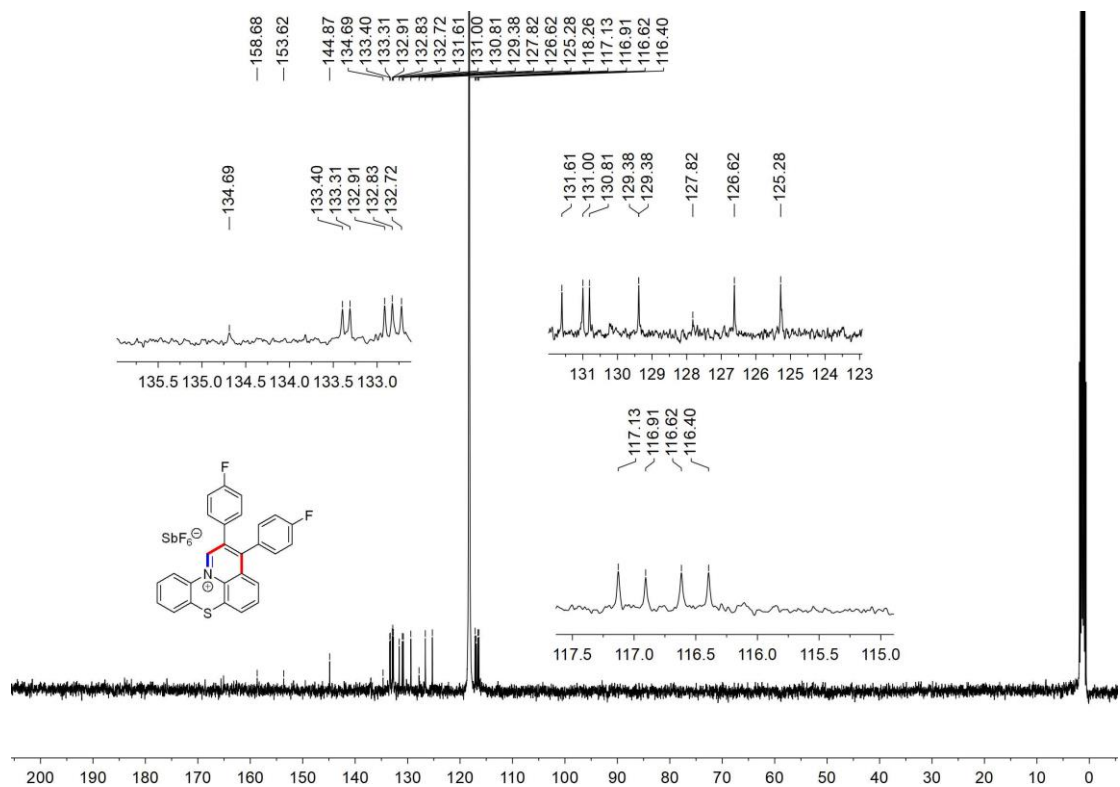

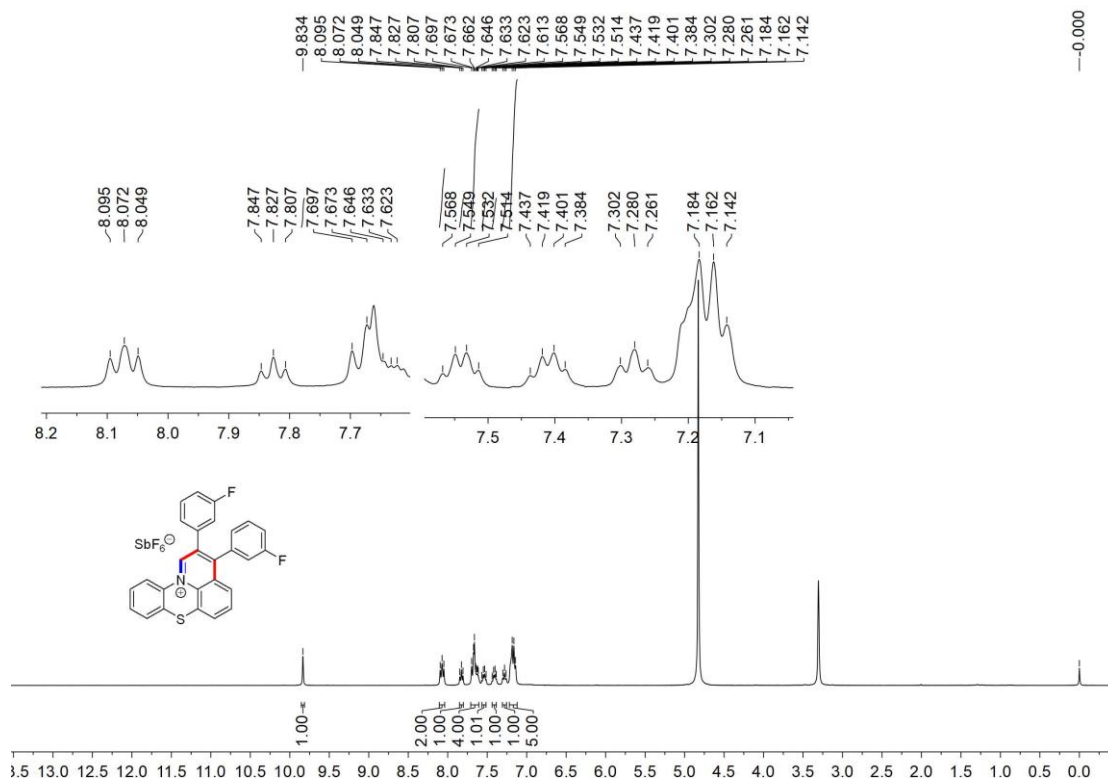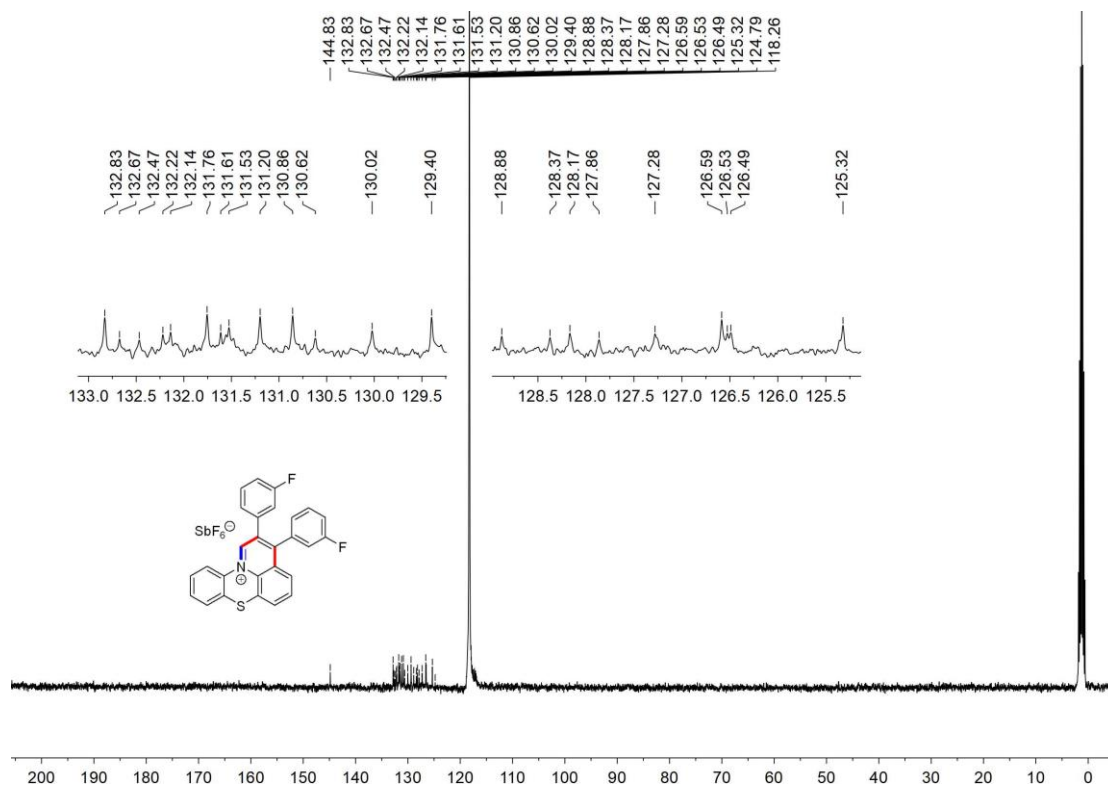



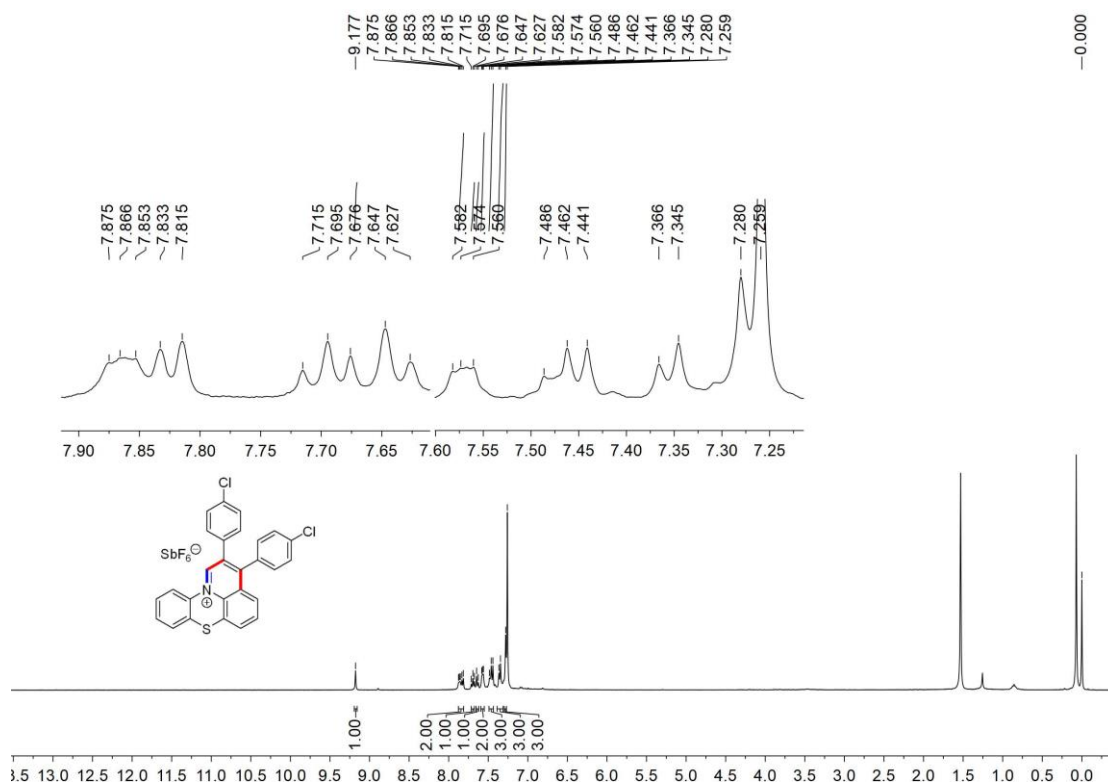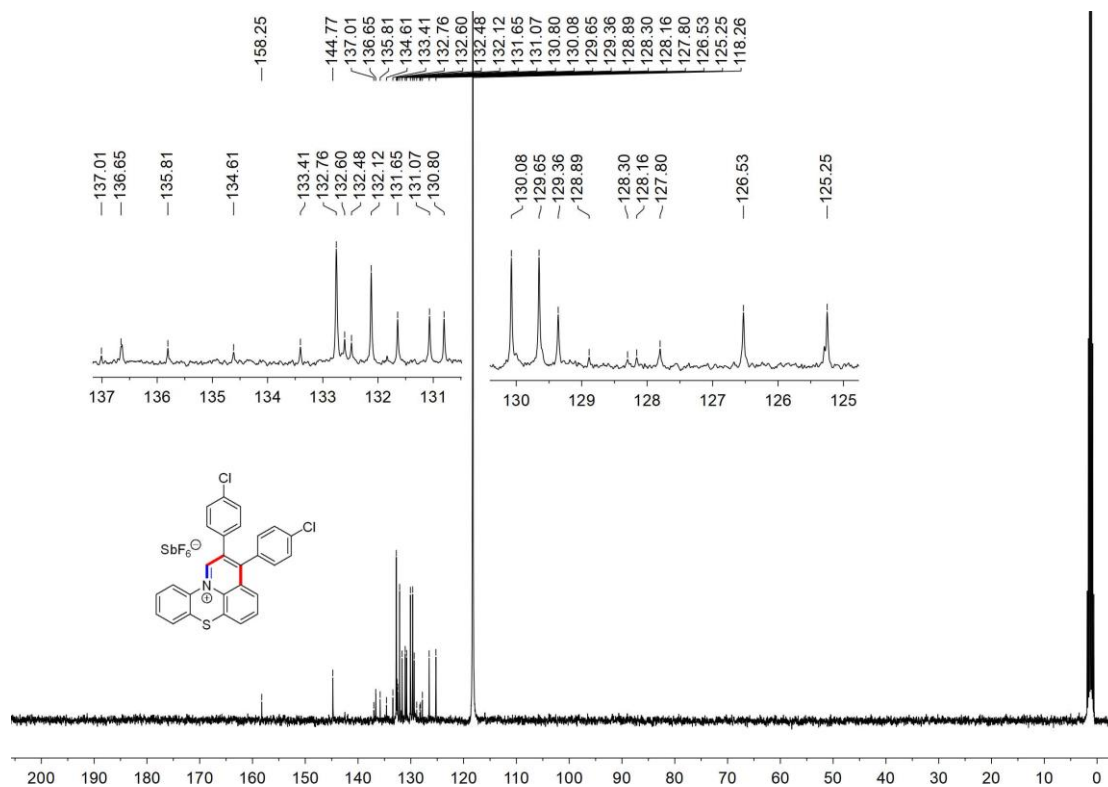

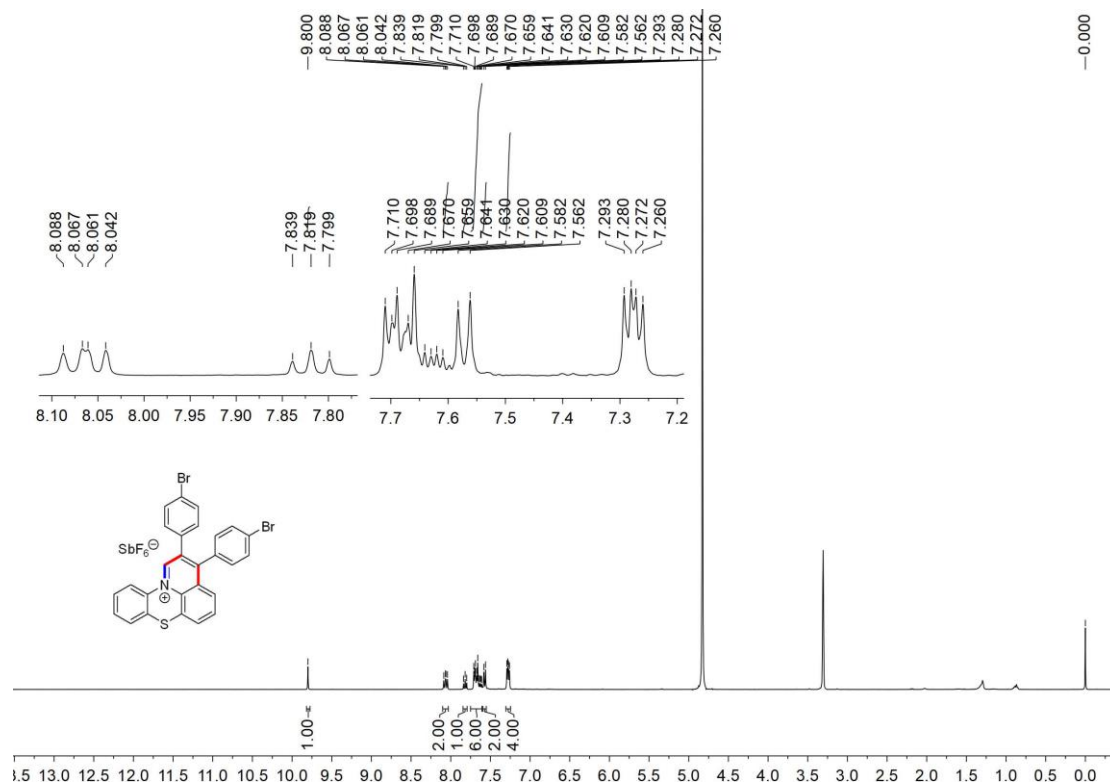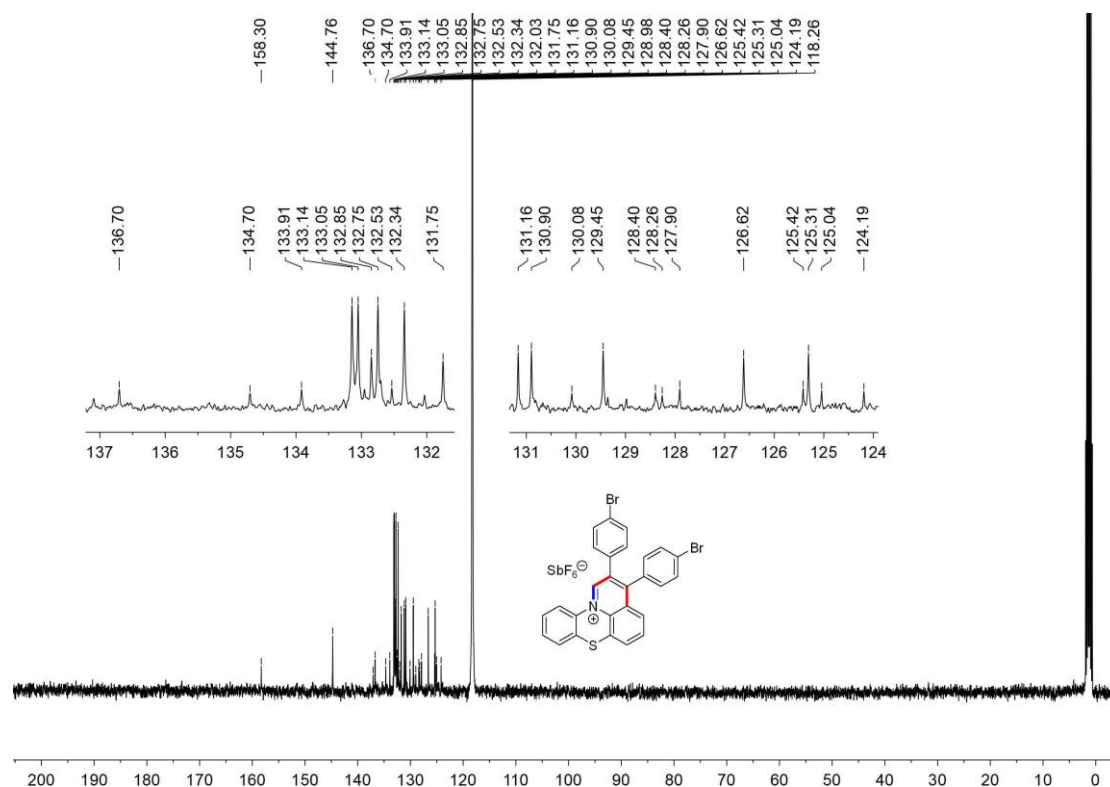

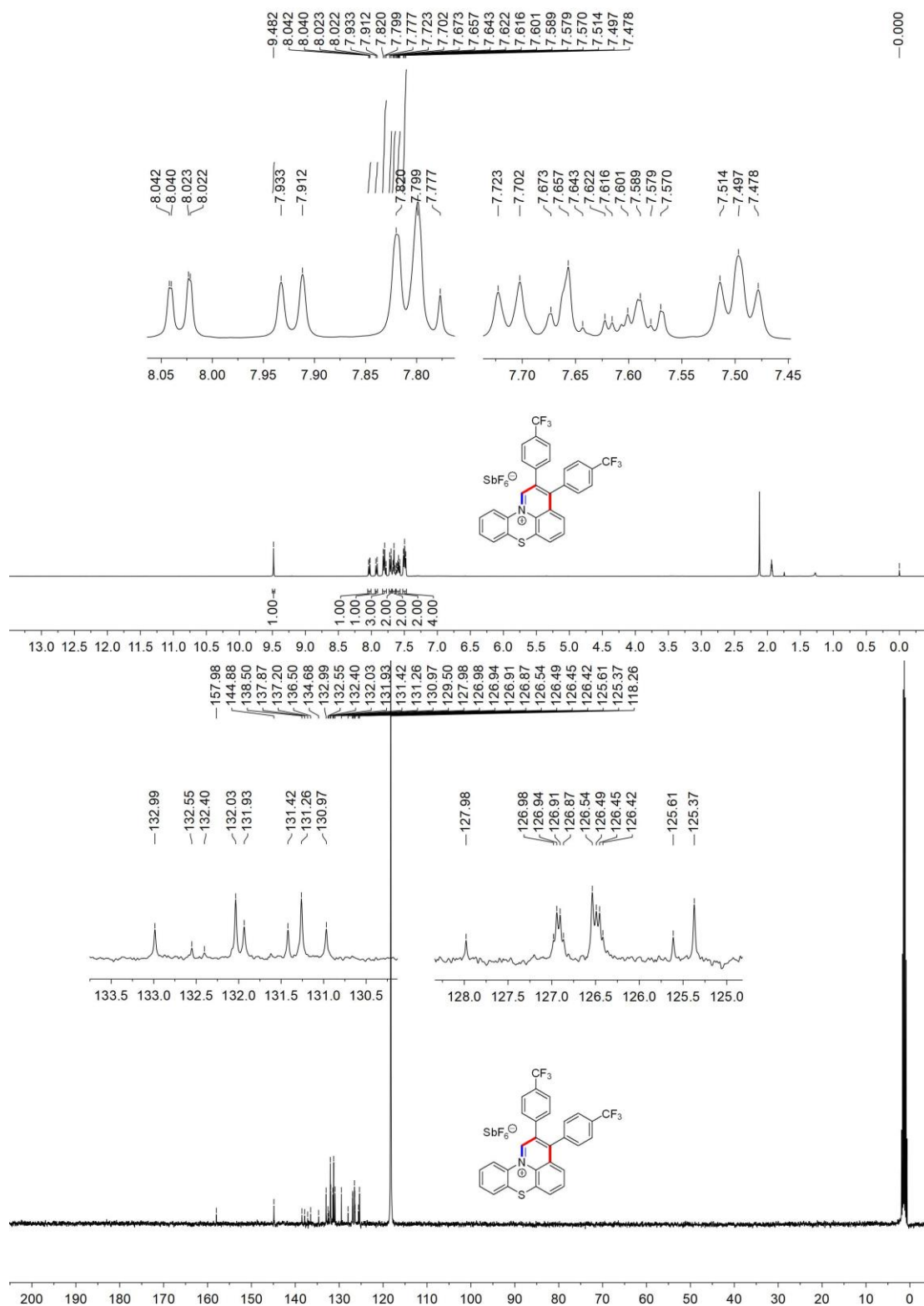

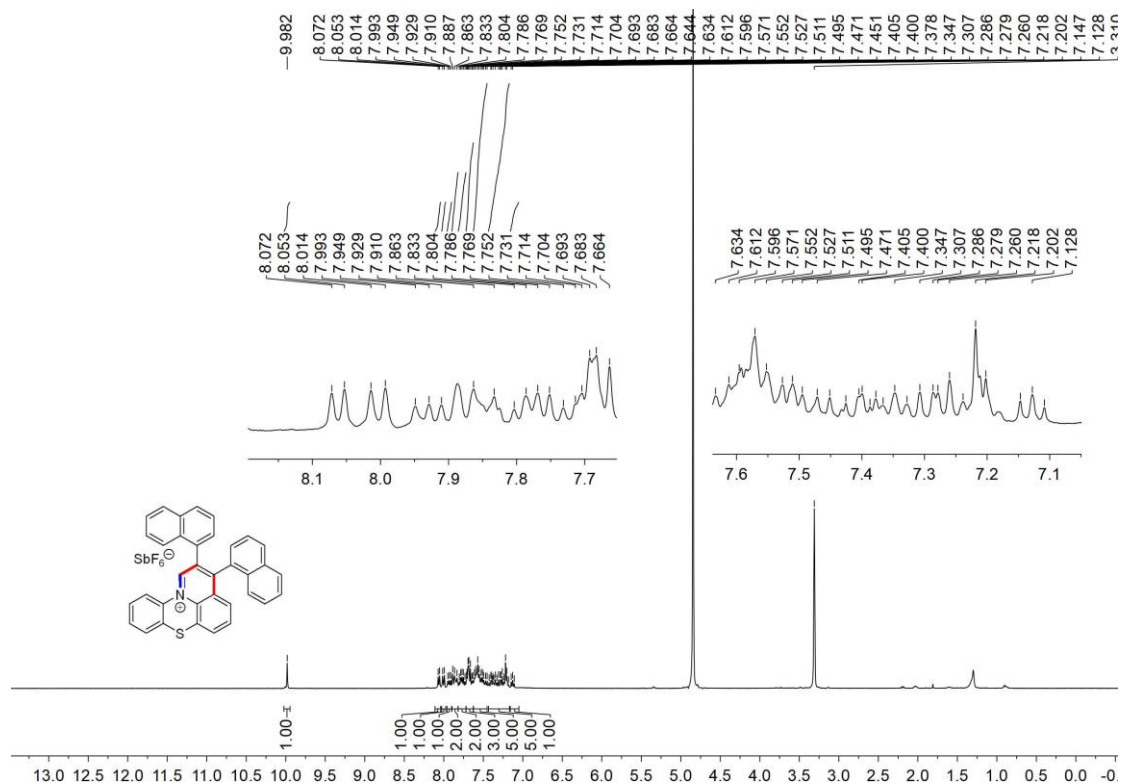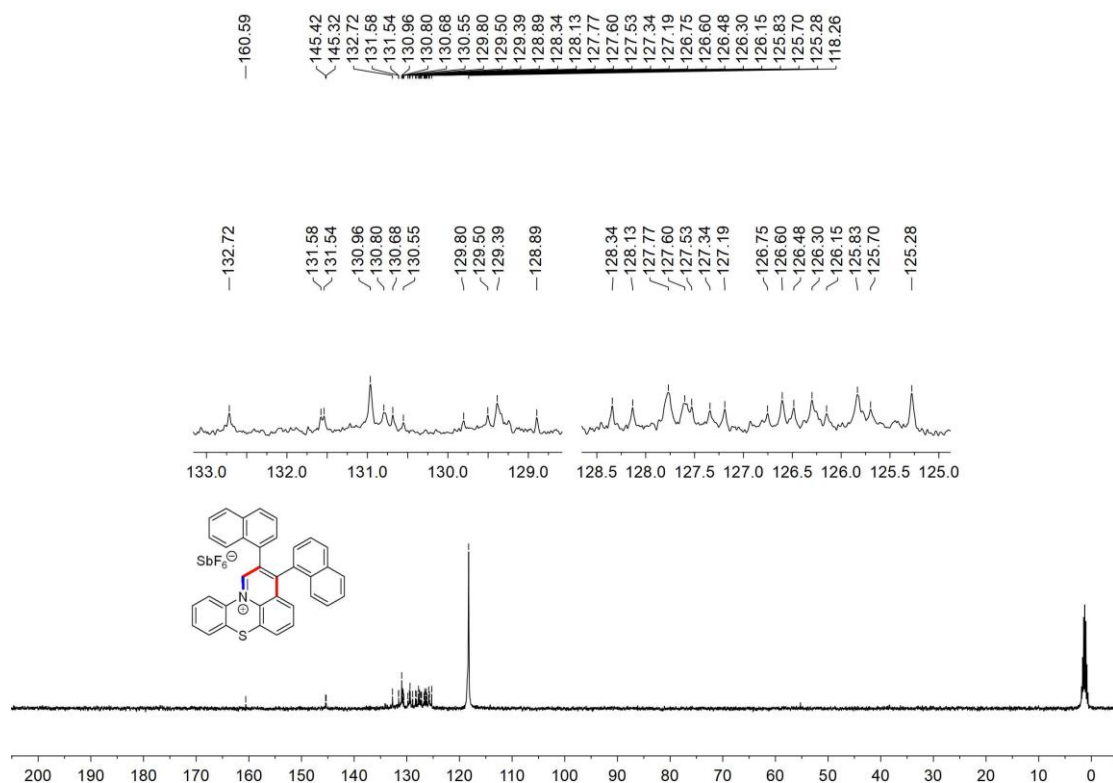

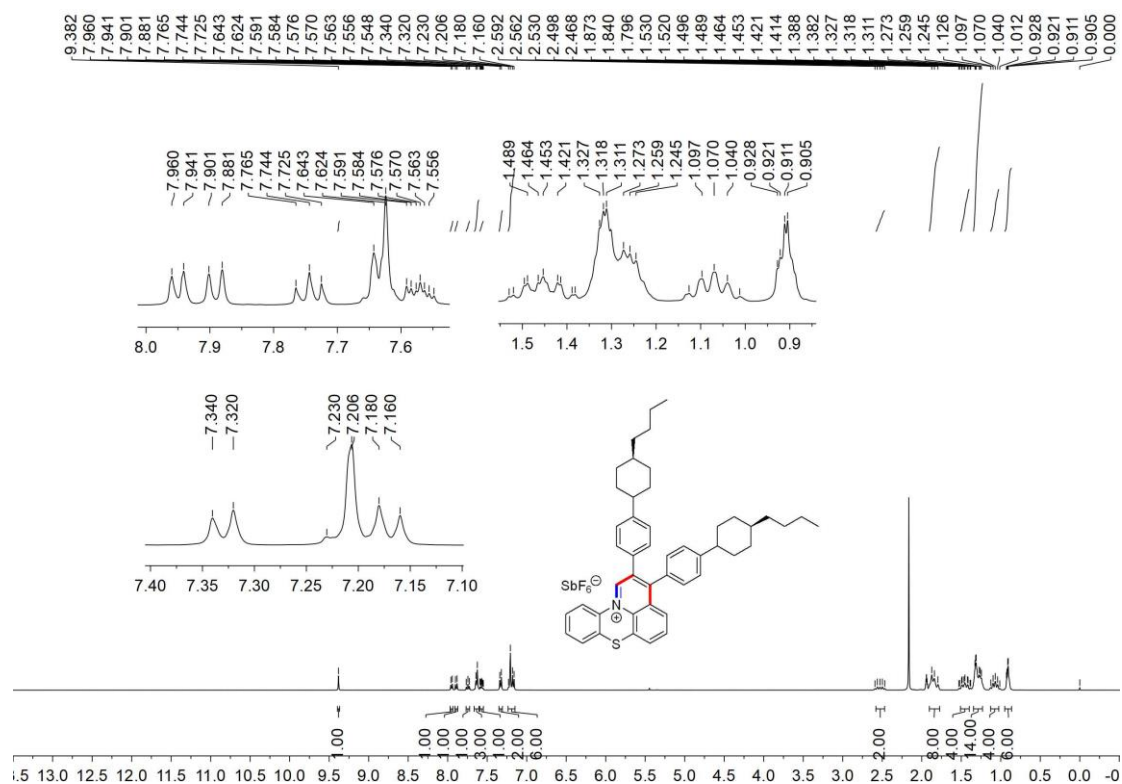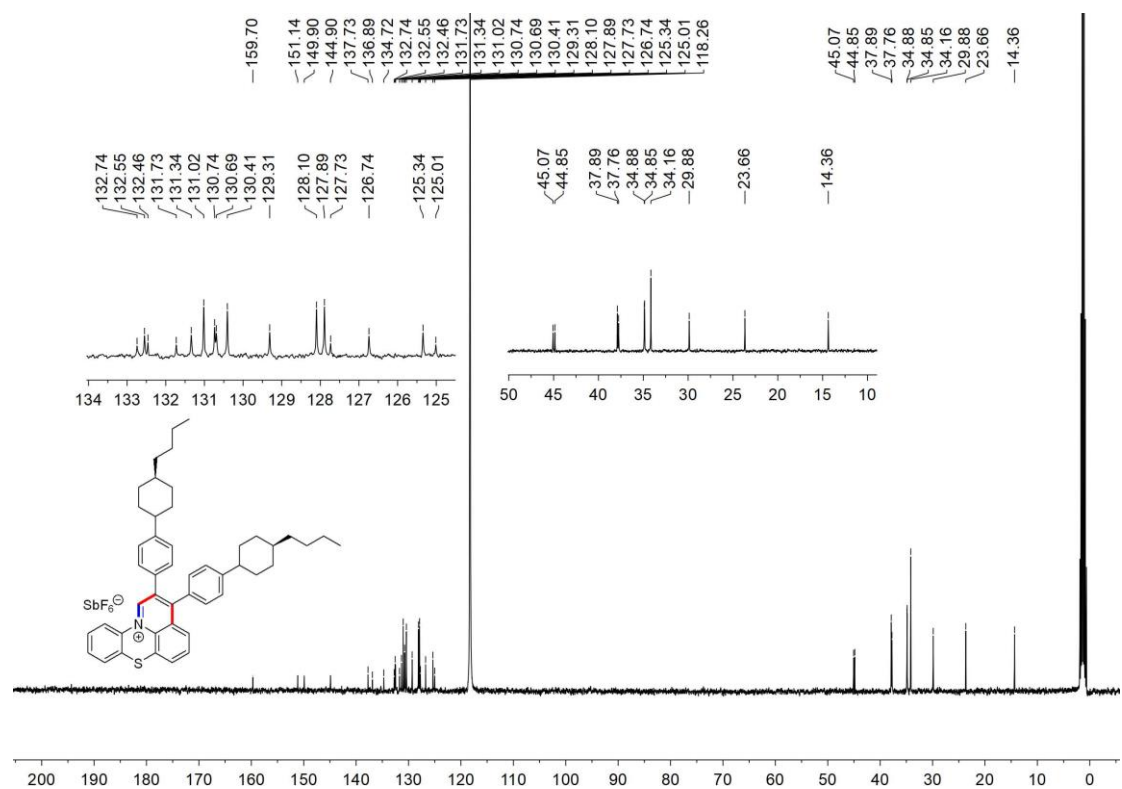

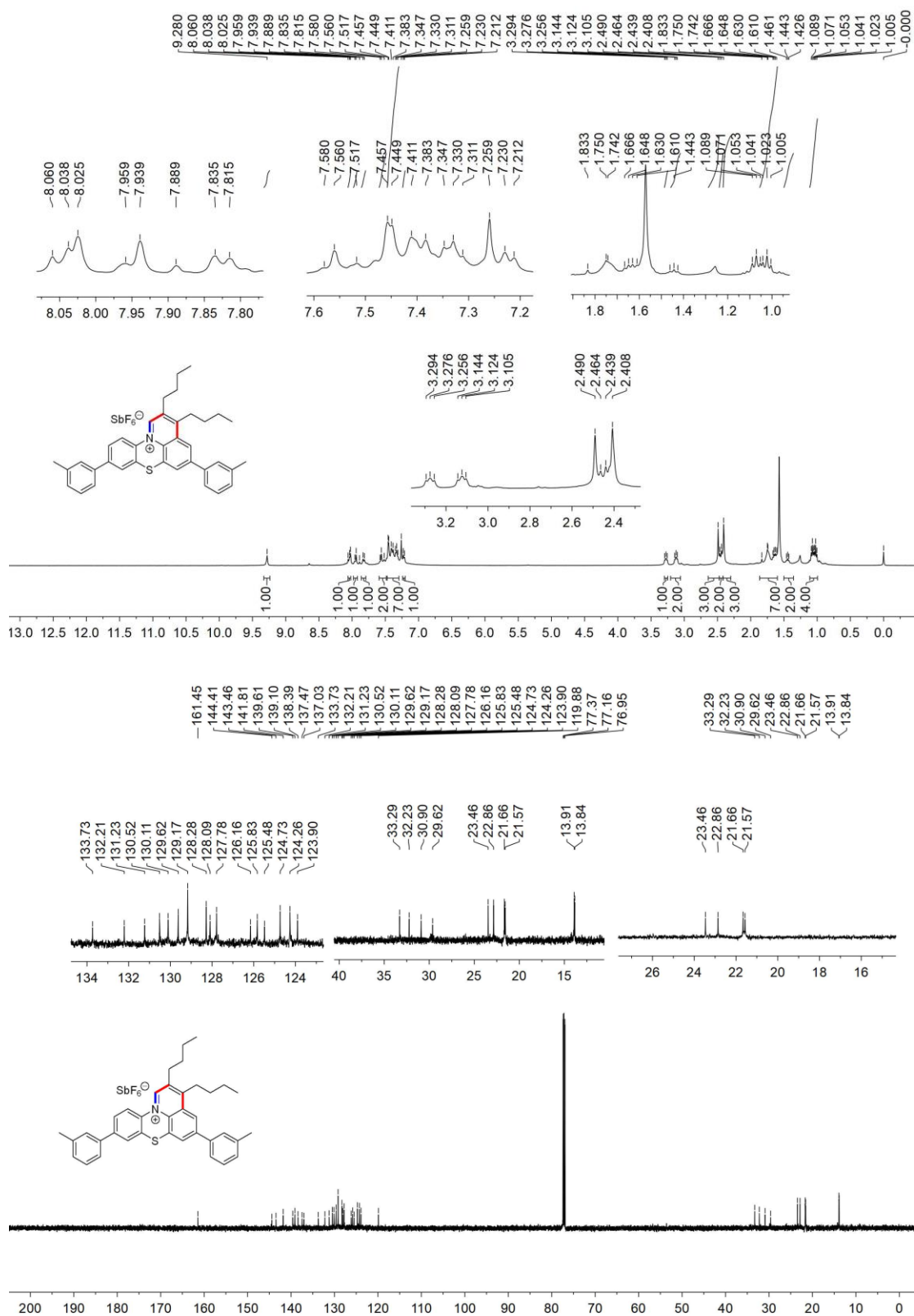

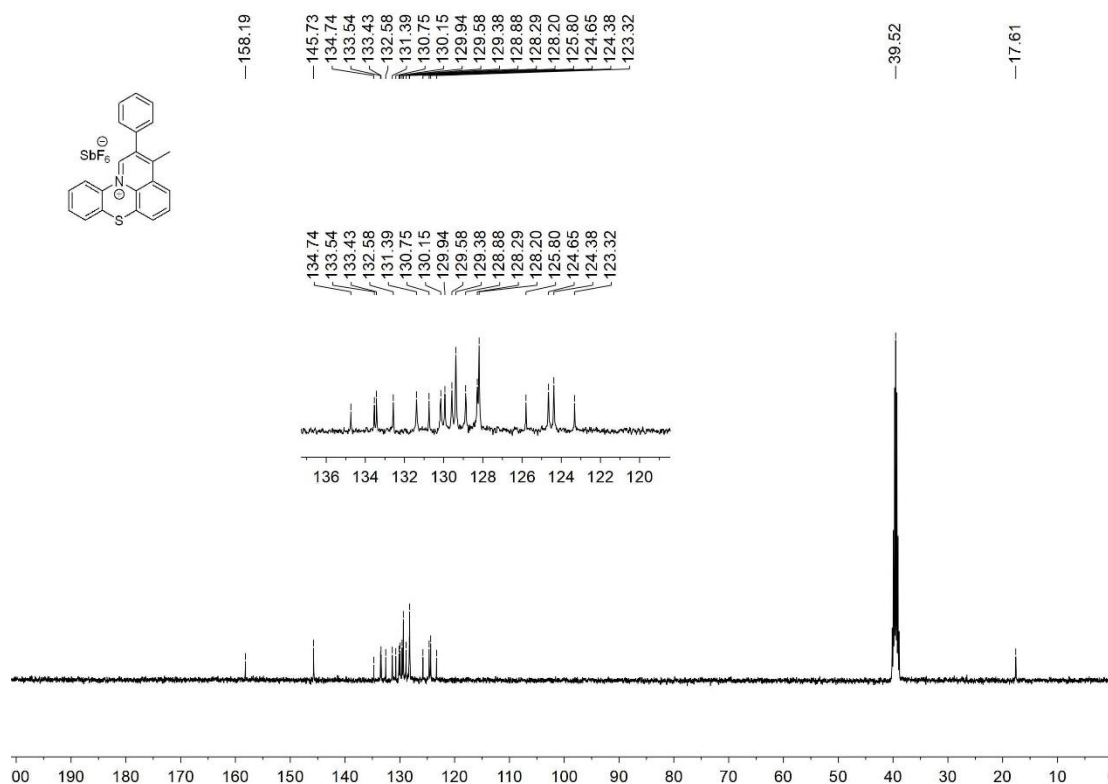

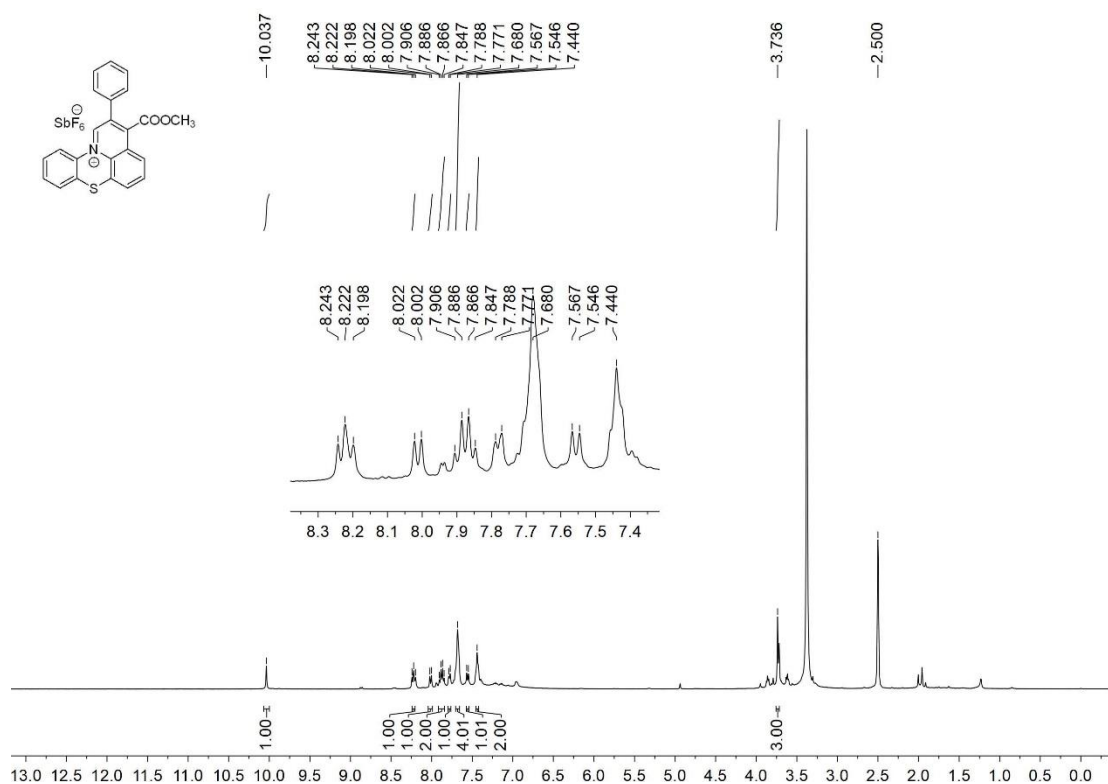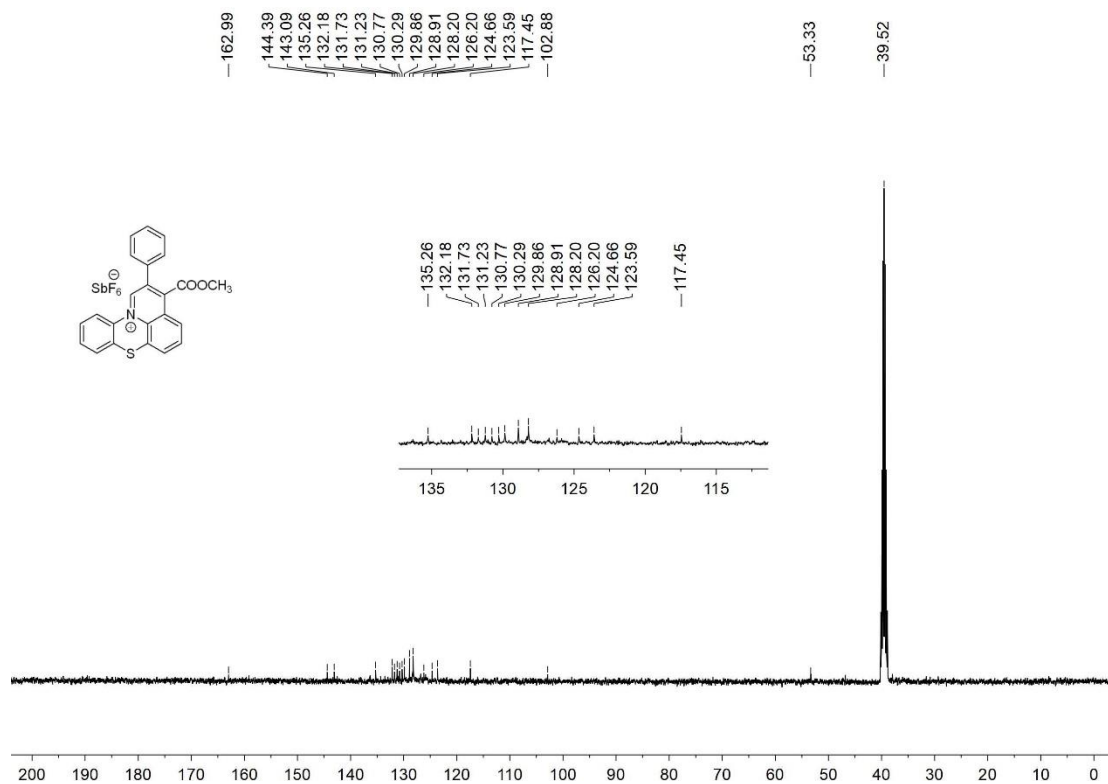

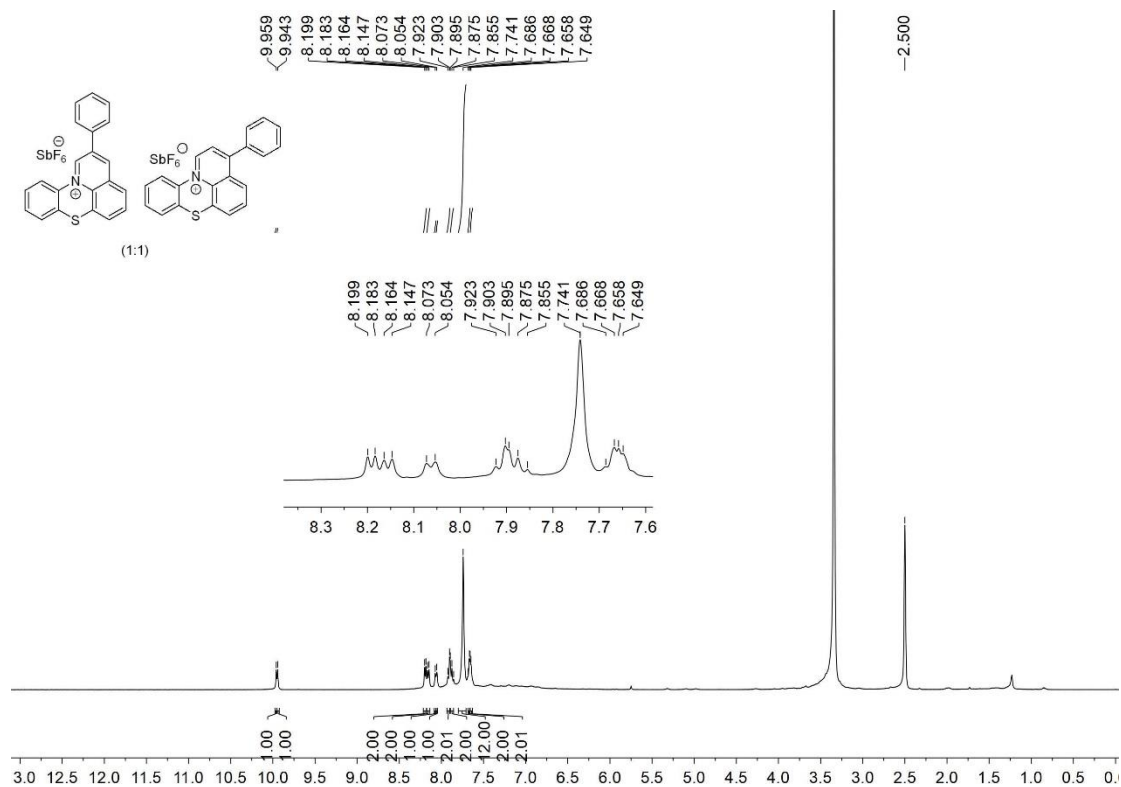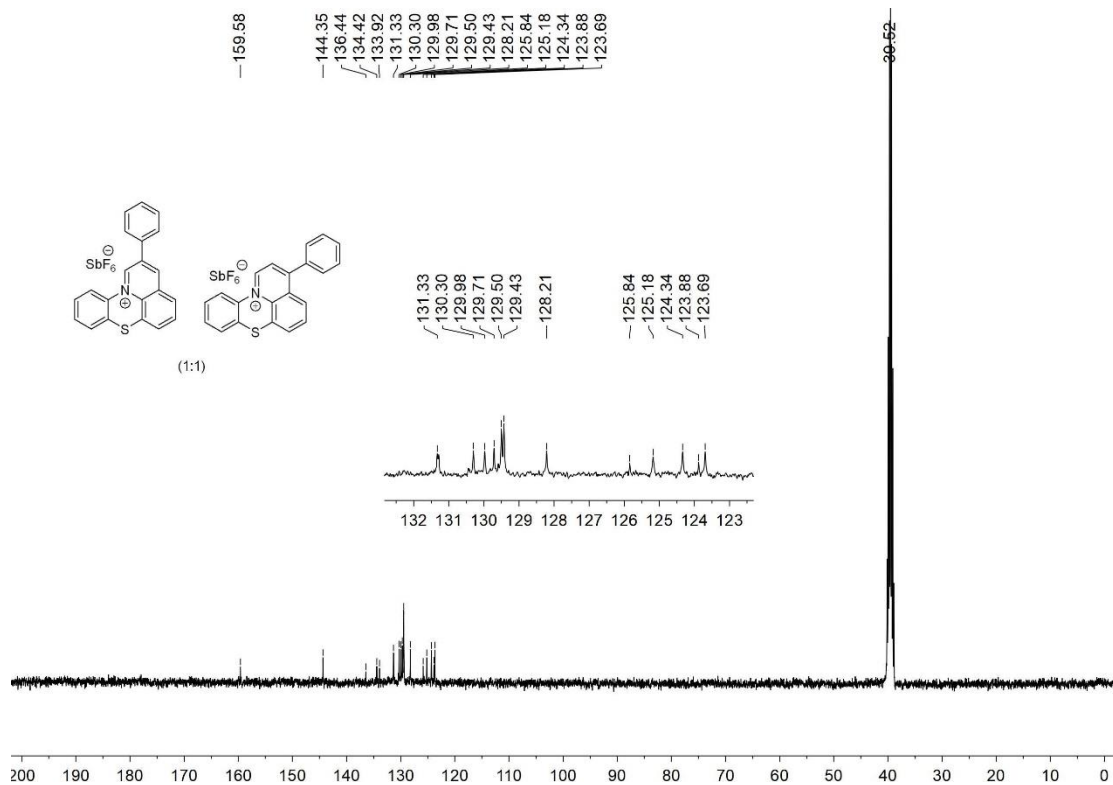

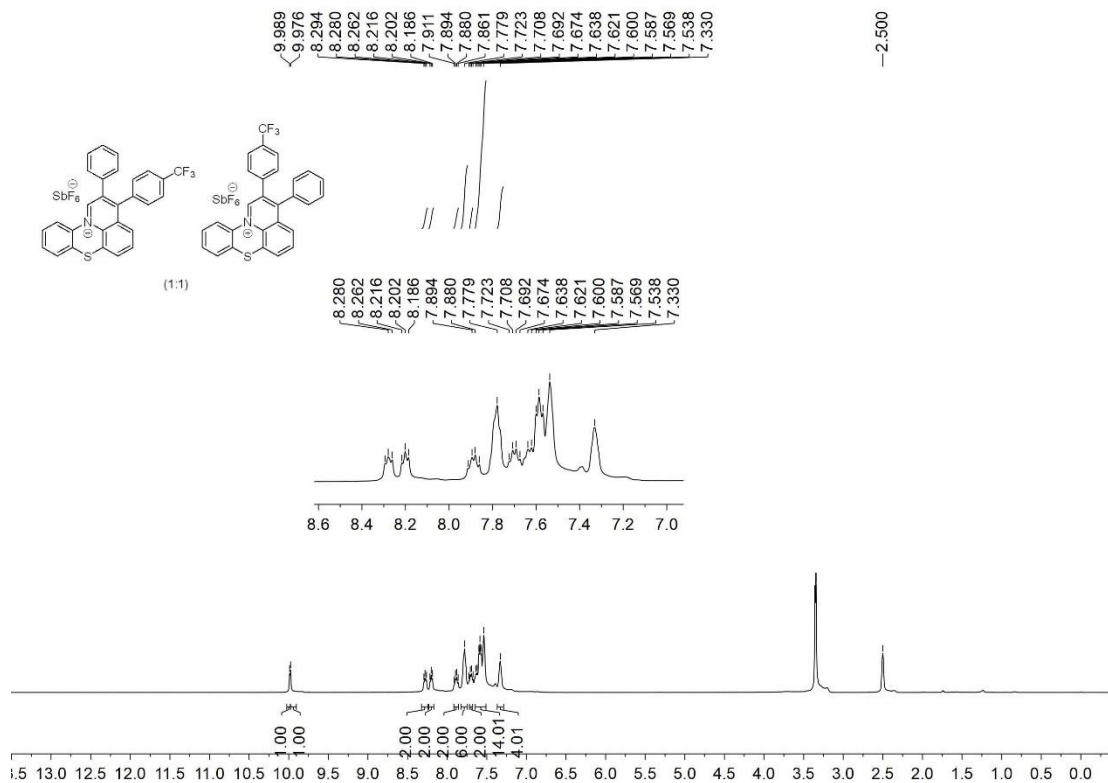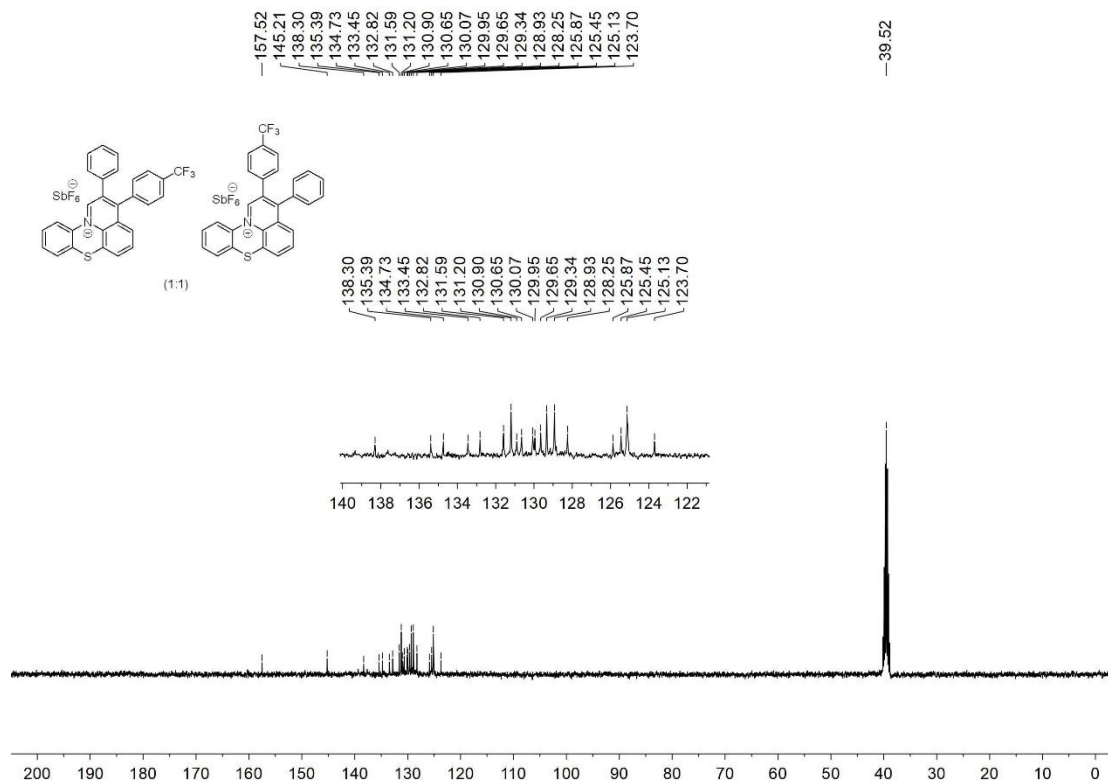



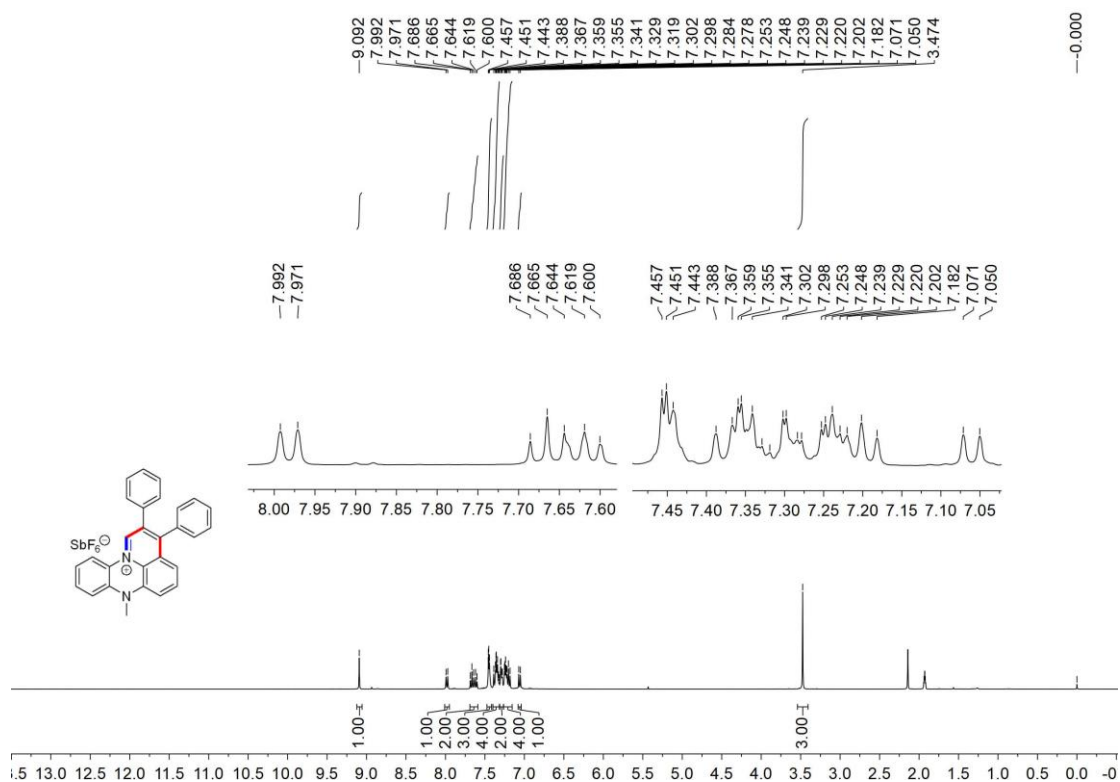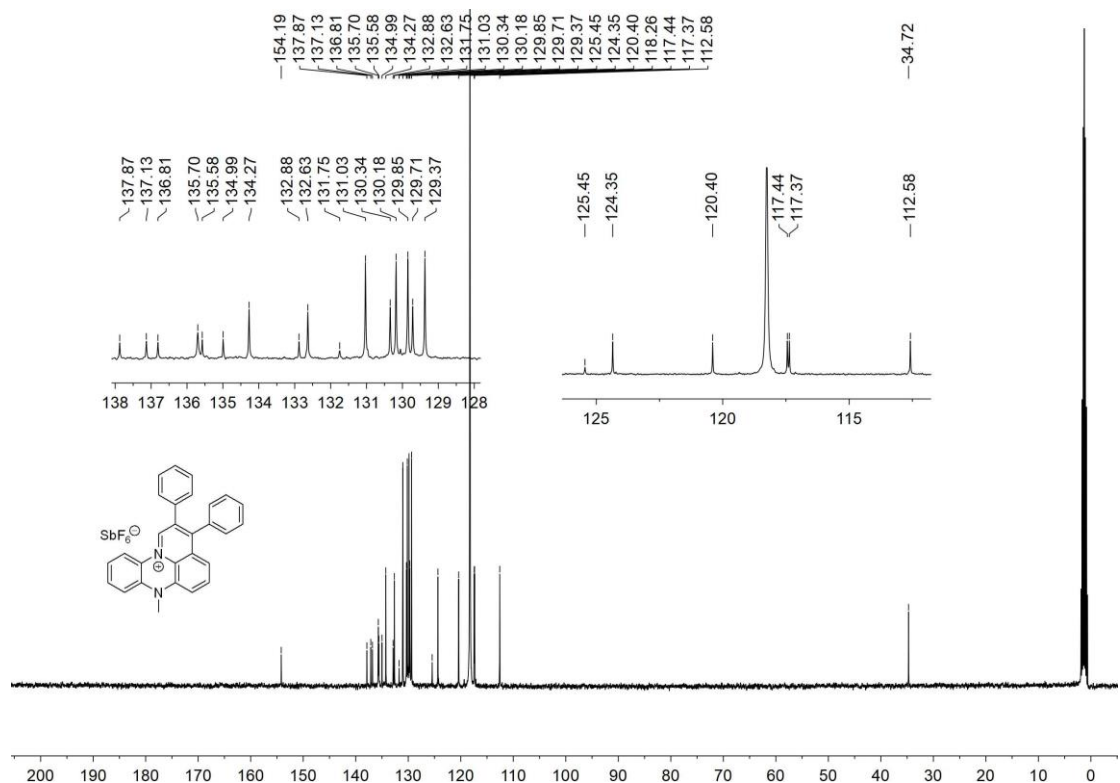



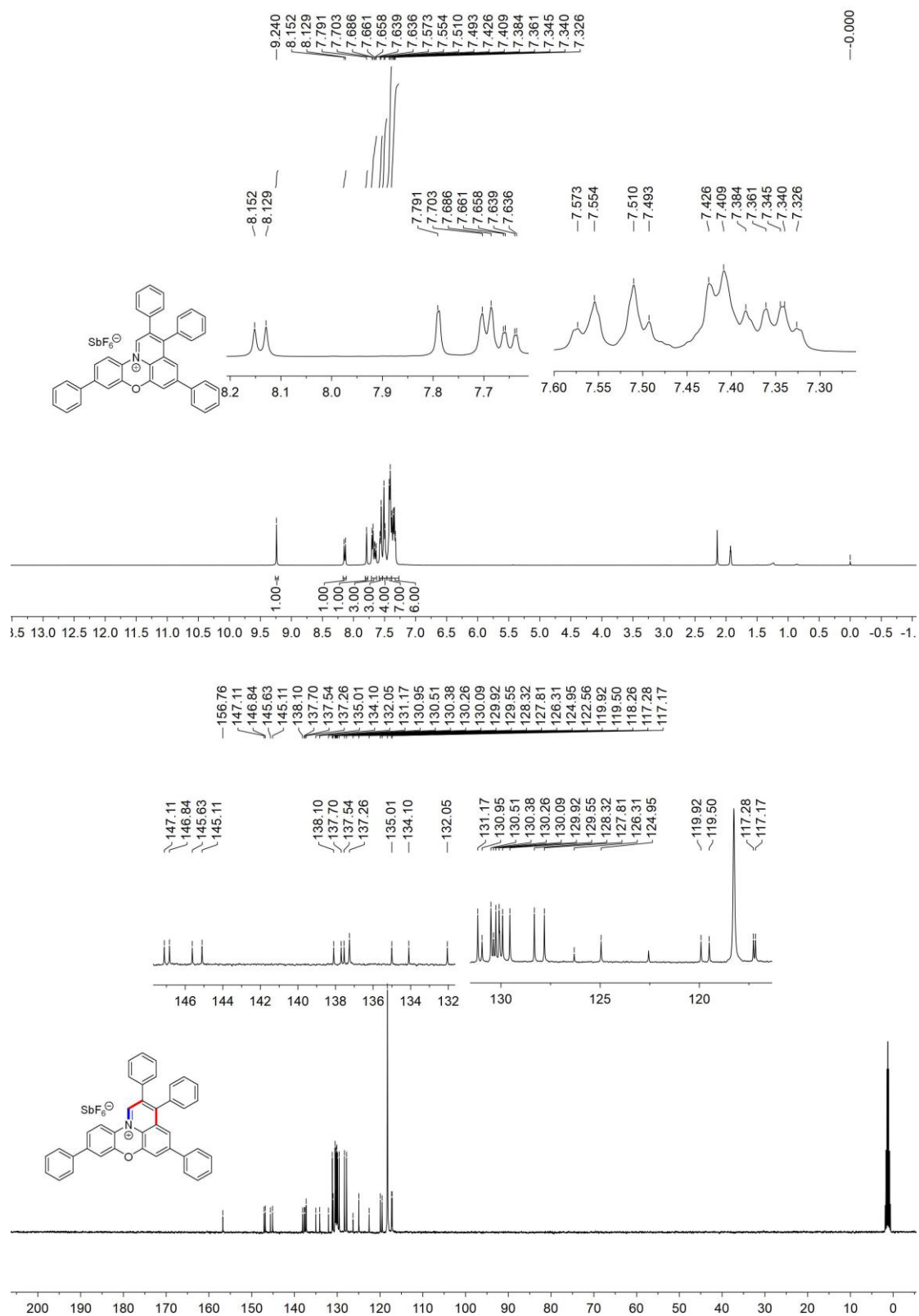

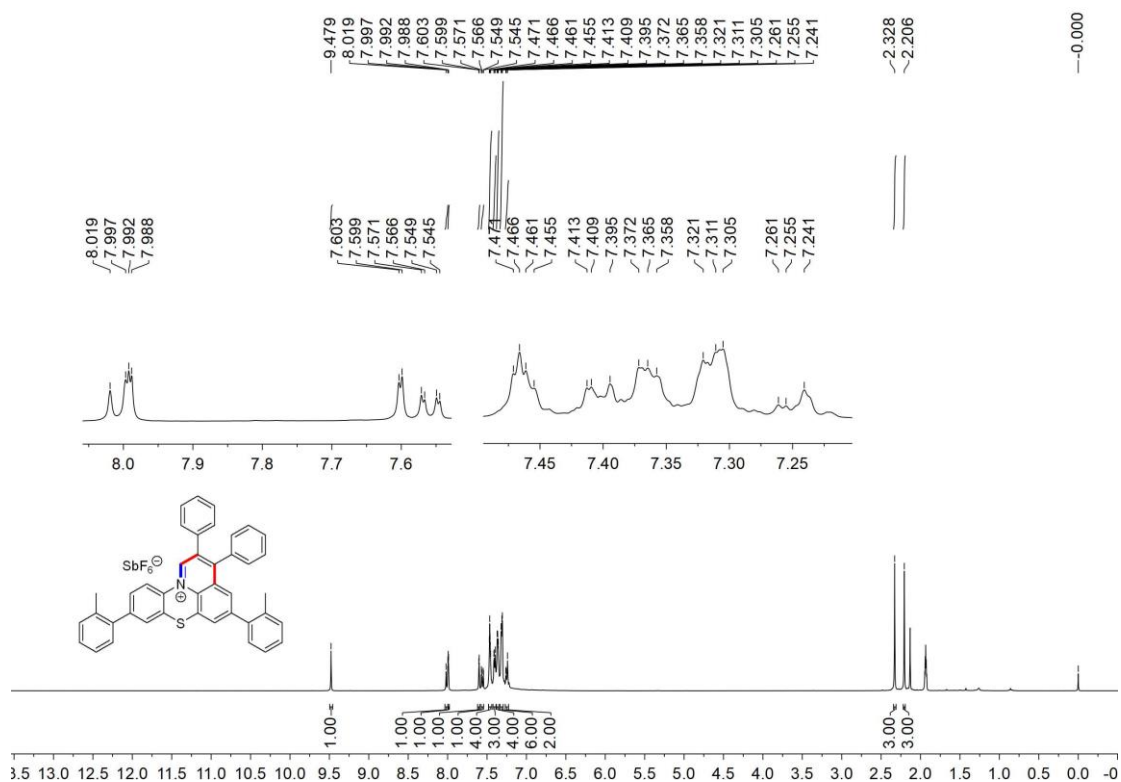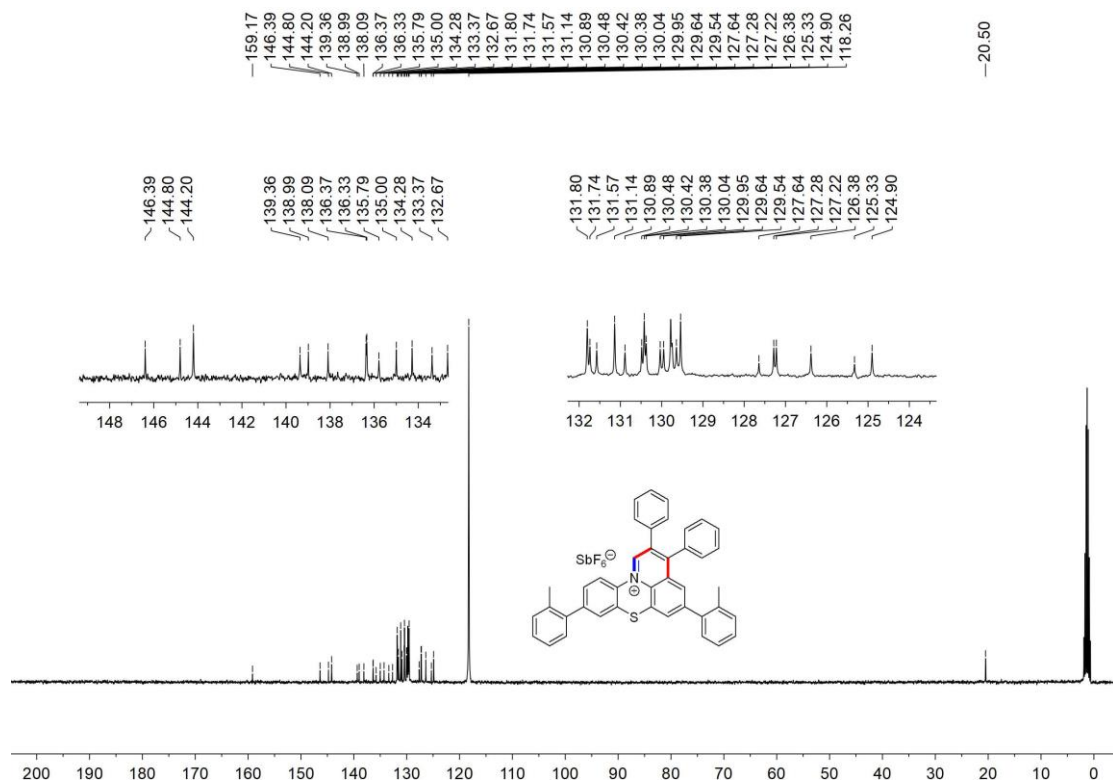



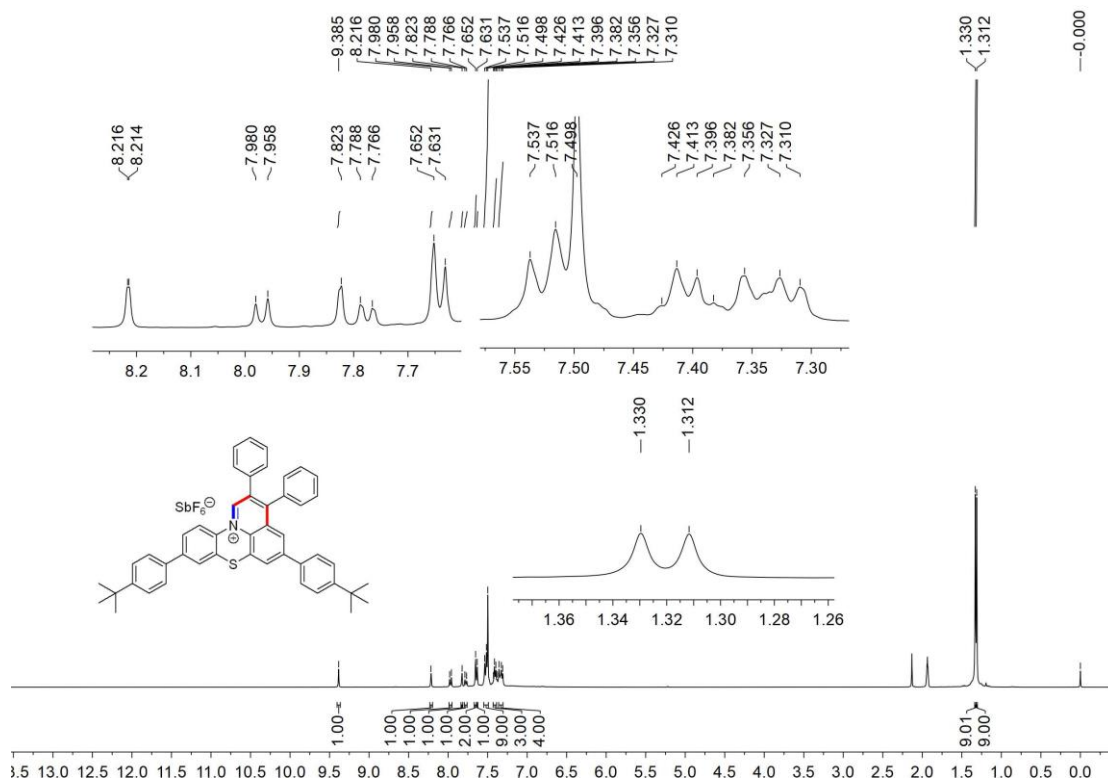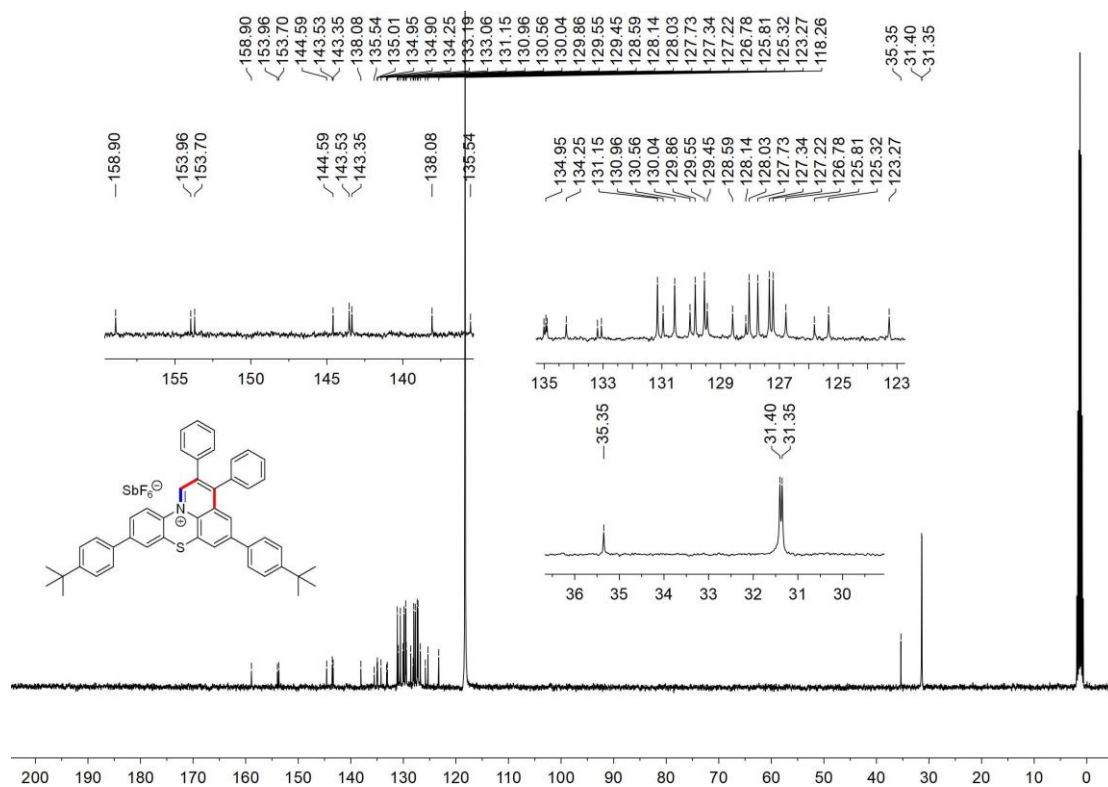







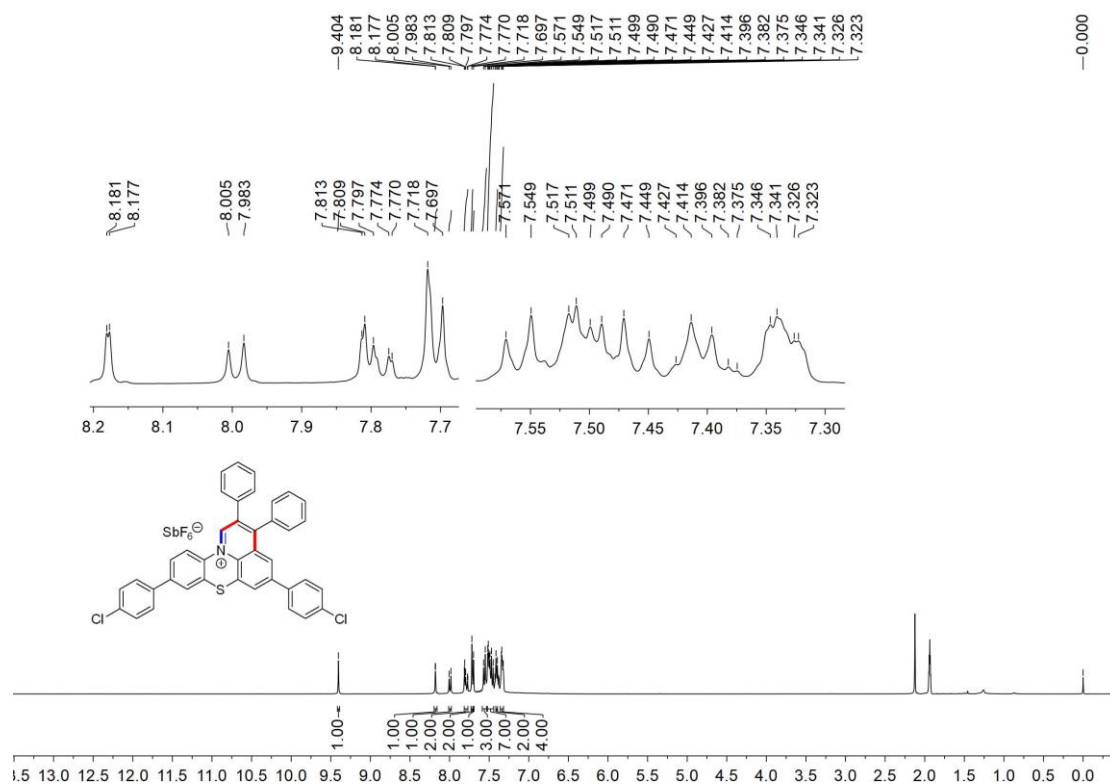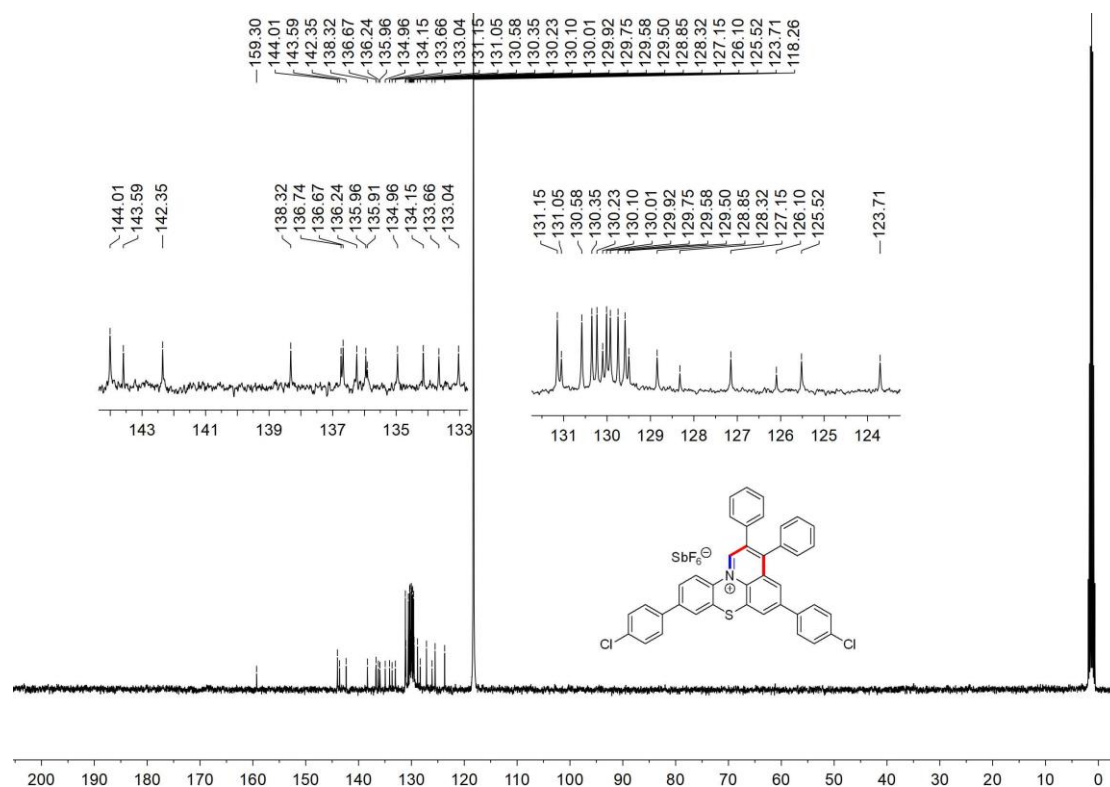

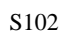



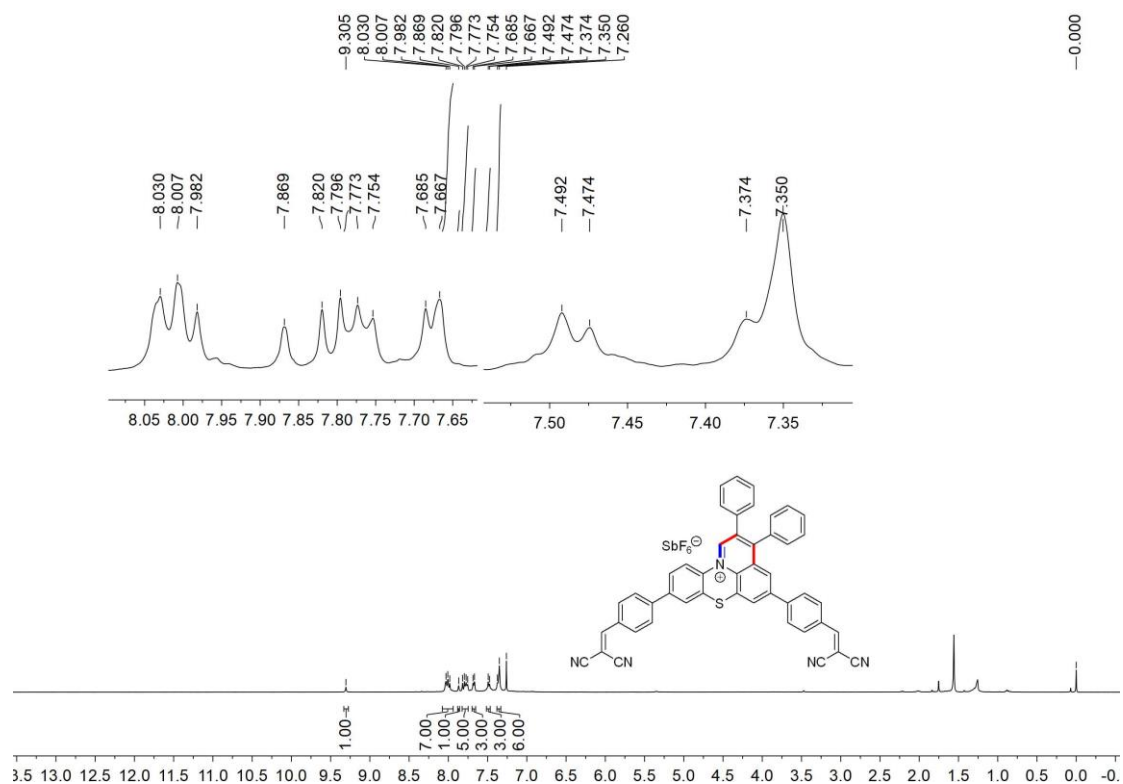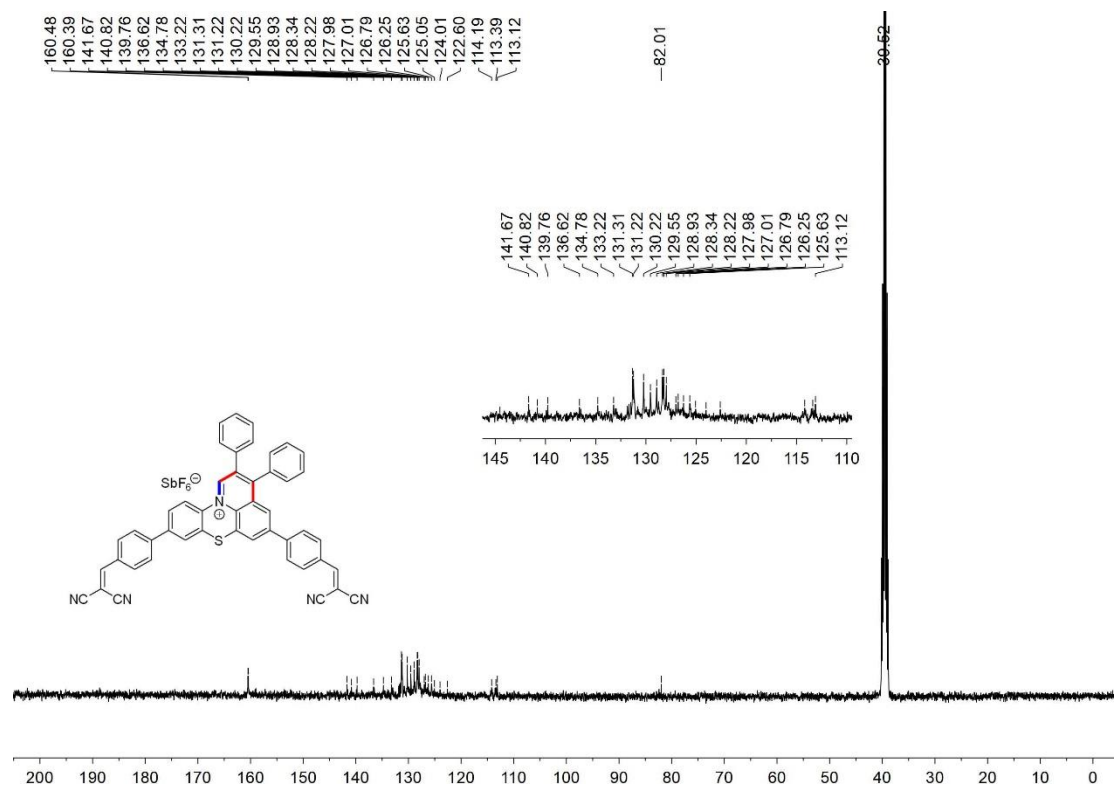



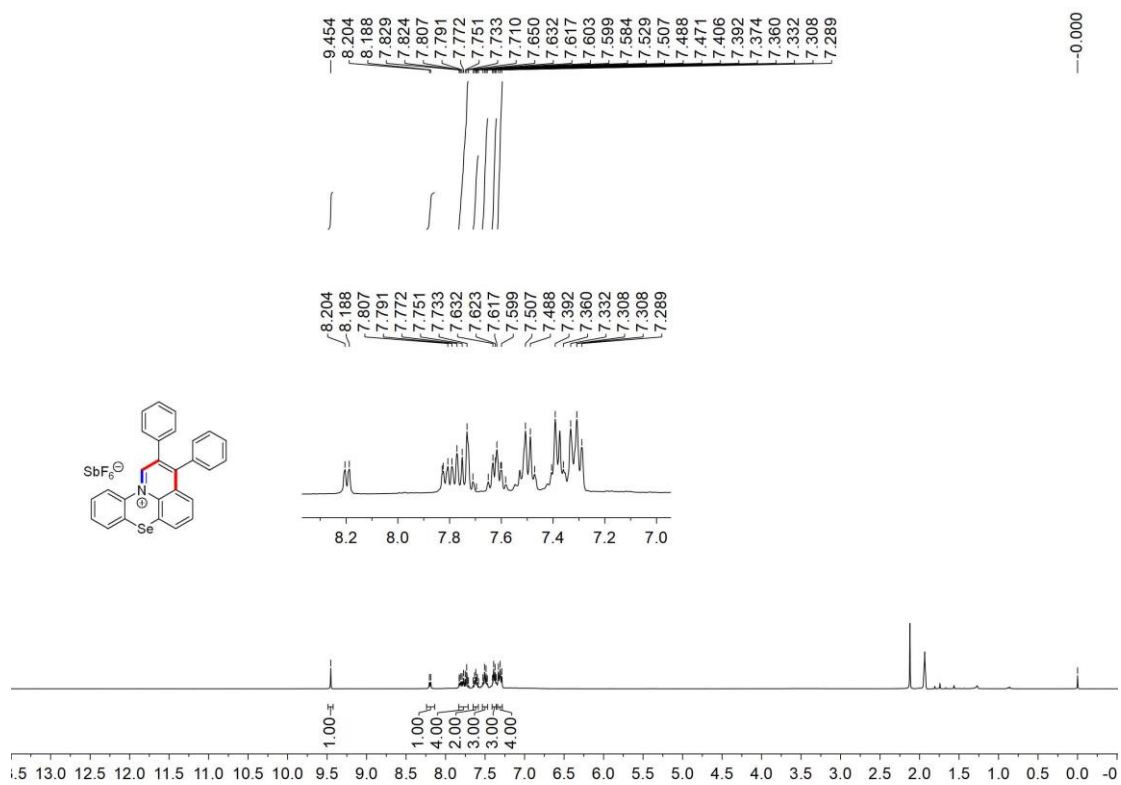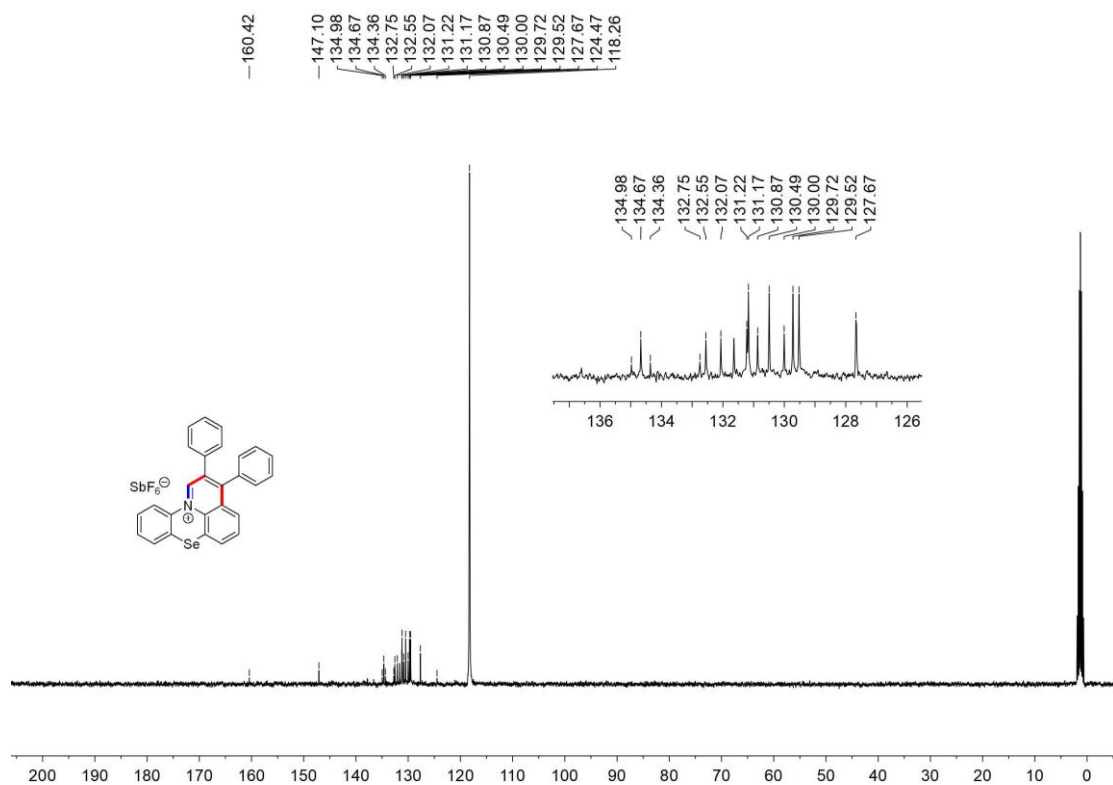



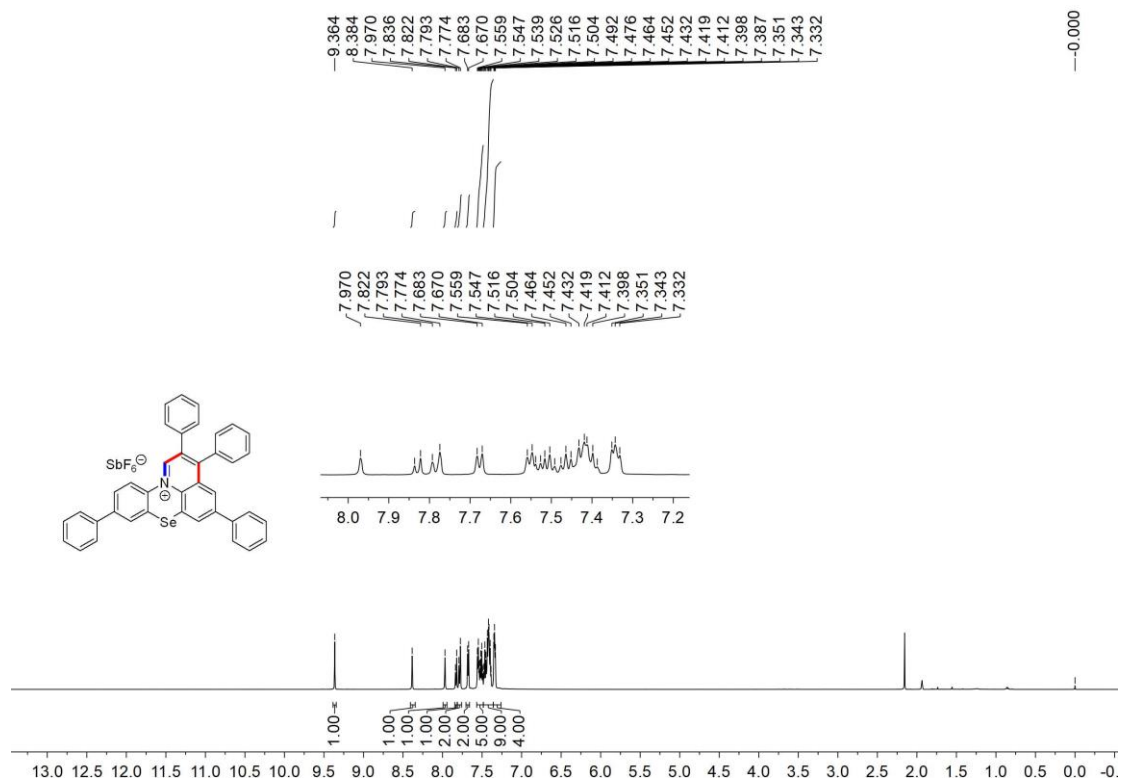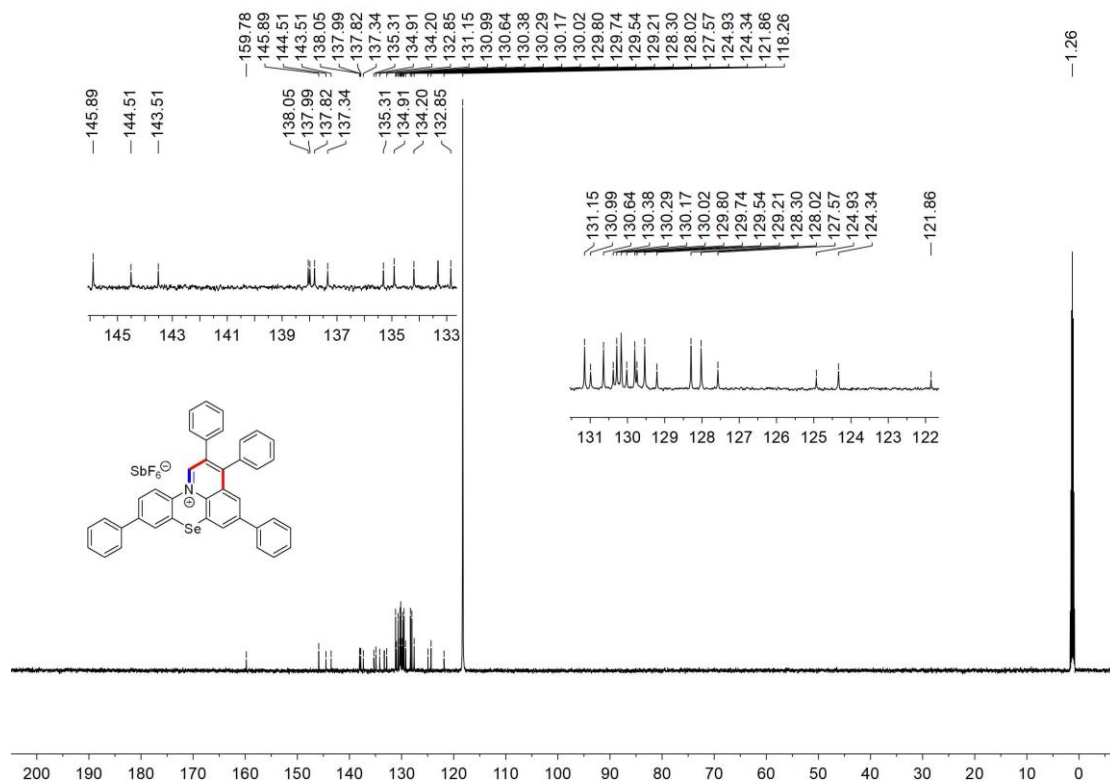

Supplement: SC-015-D4SC02188F-s001 [file SC-015-D4SC02188F-s001.pdf]
